# Oncolytic Rodent Protoparvoviruses Evade a TLR- and RLR-Independent Antiviral Response in Transformed Cells

**DOI:** 10.3390/pathogens12040607

**Published:** 2023-04-17

**Authors:** Assia Angelova, Kristina Pierrard, Claudia N. Detje, Estelle Santiago, Annabel Grewenig, Jürg P. F. Nüesch, Ulrich Kalinke, Guy Ungerechts, Jean Rommelaere, Laurent Daeffler

**Affiliations:** 1 Program Infection, Inflammation and Cancer, Clinical Cooperation Unit Virotherapy (F230), German Cancer Research Center (DKFZ), 69120 Heidelberg, Germany; a.angelova@dkfz-heidelberg.de (A.A.); guy.ungerechts@nct-heidelberg.de (G.U.); j.rommelaere@dkfz-heidelberg.de (J.R.); 2 Program Infection, Inflammation and Cancer, Division Viral Transformation Mechanisms (F030), German Cancer Research Center (DKFZ), 69120 Heidelberg, Germany; ludigs.kristina@gmail.com; 3 Institute for Experimental Infection Research, TWICNORE, Centre for Experimental and Clinical Infection Research, a Joint Venture between the Helmholtz Centre for Infection Research and the Hannover Medical School, 30625 Hannover, Germany; twincore@twincore.de (C.N.D.); kalinke.ulrich@mh-hannover.de (U.K.); 4 CNRS, IPHC UMR 7178, Université de Strasbourg, F-67000 Strasbourg, France; estelle.santiago@iphc.cnrs.fr; 5 Program Infection, Inflammation and Cancer, Division DNA Vectors (F160), German Cancer Research Center (DKFZ), 69120 Heidelberg, Germany; a.grewenig@dkfz-heidelberg.de; 6 Program Infection, Inflammation and Cancer, Division Virus-Associated Carcinogenesis (F170), German Cancer Research Center (DKFZ), 69120 Heidelberg, Germany; jpf.nuesch@dkfz-heidelberg.de; 7 Department of Medical Oncology, National Center for Tumor Diseases (NCT), Heidelberg University Hospital, 69120 Heidelberg, Germany

**Keywords:** parvovirus, oncolytic, innate immune response, type-I interferons, cancer cells, innate immune response evasion mechanism

## Abstract

The oncolytic rodent protoparvoviruses (PVs) minute virus of mice (MVMp) and H-1 parvovirus (H-1PV) are promising cancer viro-immunotherapy candidates capable of both exhibiting direct oncolytic activities and inducing anticancer immune responses (AIRs). Type-I interferon (IFN) production is instrumental for the activation of an efficient AIR. The present study aims at characterizing the molecular mechanisms underlying PV modulation of IFN induction in host cells. MVMp and H-1PV triggered IFN production in semi-permissive normal mouse embryonic fibroblasts (MEFs) and human peripheral blood mononuclear cells (PBMCs), but not in permissive transformed/tumor cells. IFN production triggered by MVMp in primary MEFs required PV replication and was independent of the pattern recognition receptors (PRRs) Toll-like (TLR) and RIG-like (RLR) receptors. PV infection of (semi-)permissive cells, whether transformed or not, led to nuclear translocation of the transcription factors NFĸB and IRF3, hallmarks of PRR signaling activation. Further evidence showed that PV replication in (semi-)permissive cells resulted in nuclear accumulation of dsRNAs capable of activating mitochondrial antiviral signaling (MAVS)-dependent cytosolic RLR signaling upon transfection into naïve cells. This PRR signaling was aborted in PV-infected neoplastic cells, in which no IFN production was detected. Furthermore, MEF immortalization was sufficient to strongly reduce PV-induced IFN production. Pre-infection of transformed/tumor but not of normal cells with MVMp or H-1PV prevented IFN production by classical RLR ligands. Altogether, our data indicate that natural rodent PVs regulate the antiviral innate immune machinery in infected host cells through a complex mechanism. In particular, while rodent PV replication in (semi-)permissive cells engages a TLR-/RLR-independent PRR pathway, in transformed/tumor cells this process is arrested prior to IFN production. This virus-triggered evasion mechanism involves a viral factor(s), which exert(s) an inhibitory action on IFN production, particularly in transformed/tumor cells. These findings pave the way for the development of second-generation PVs that are defective in this evasion mechanism and therefore endowed with increased immunostimulatory potential through their ability to induce IFN production in infected tumor cells.

## 1. Introduction

Oncolytic viruses (OVs) replicate (oncotropism) and trigger lysis (oncolysis) preferentially in malignant cells, leaving normal/healthy cells unharmed [1]. In addition to their direct oncolytic activity, the major and most attractive aspect of OVs regarding anticancer therapies resides in their capacity to elicit both innate and adaptive immune responses against neoplastic cells, acting thereby like a cancer vaccine triggering immunotherapeutic effects [2,3,4]. The innate immune system is the first line of defense of organisms against infections and is crucial for detecting and clearing pathogenic invaders. Indeed, upon virus (including OV) infection, host cells produce/expose host factors termed DAMPs (damage-associated molecular patterns), here including adenosine triphosphate (ATP), cell-free nucleic acids, calreticulin, heat shock proteins (HSPs), type-I interferons (IFNs) α and β and high mobility group box 1 (HMGB1) protein, that are sensed by immune cells and lead to their priming and/or activation [5,6]. Another type of immunogenic factor produced by OV-infected cells are viral elements (mainly dsRNAs, ssRNAs and virus genomes), which accumulate in infected cells upon virus replication and multiplication. These viral elements are released in the extracellular milieu at the time of cell lysis [7] and qualified as pathogen-associated molecular patterns (PAMPs). Similar to DAMPs, PAMPs are potent activators of an anticancer immune response [8]. In order to exert their immunogenic effects, PAMPs and DAMPs need to bind receptors present on the surface of, or within (cytoplasm), neighboring cells including immune cells, such as dendritic cells (DCs), natural killer (NK) cells and T and B lymphocytes. These receptors are germline-encoded and known as pattern recognition receptors (PRRs) [9,10,11]. PRRs belong presently to five different families, namely, membranous Toll-like receptors (TLRs), C-type lectin receptors (CLRs), cytosolic RIG-like receptors (RLRs), DNA sensors (cGAS, IFI16, DAI, etc.) and Nod-like receptors (NLRs) [7,12,13,14,15]. Recently, cGAS and IFI16 were shown to be also present in the cell nucleus, where they are suggested to sense infections with DNA viruses (e.g., herpes simplex virus type 1) [16,17,18]. The engagement of the above mentioned PRRs with PAMPs and DAMPs leads to the intracellular activation of signaling pathways that trigger the nuclear translocation of transcription factors, such as nuclear factor kappa B (NFκB) and interferon regulatory factors (IRF) 3 and 7. Upon binding to promoter regions of the host cell DNA, these factors induce the production of pro-inflammatory cytokines, chemokines and type-I interferons (IFN-α and -β) that enter a complex cooperation to control infection [7,14,19].

In particular, type-I IFNs produced by all vertebrate nucleated cells (IFN-β) and mainly by plasmacytoid dendritic cells (pDCs) (IFN-α) play a pivotal role in the development of an efficient antiviral response, in addition to their well-known anti-proliferative effects [20]. Once released from infected cells, these cytokines bind, in a paracrine and autocrine fashion, to virtually ubiquitously expressed cell-surface heterodimeric receptors (IFNARs) [21]. IFNAR engagement leads to the downstream activation of canonical as well as non-canonical signaling pathways that regulate the transcription of numerous genes. The canonical pathway (JAK–STAT pathway) consists in the cytoplasmic phosphorylation and dimerization of STAT_1_ and STAT_2_ and their association to IRF9 to form a heterotrimeric transcriptional complex (ISGF3) that penetrates the nucleus [22]. In the nucleus, ISGF3 *trans*-activates IFN-stimulated responsive elements (ISREs) present in promoters of interferon-stimulated genes (ISGs) both in infected as well as in adjacent non-infected cells. This process leads to the expression of hundreds of ISG proteins including PKR (double-stranded RNA-dependent protein kinase), OAS (2′,5′-oligoadenylate synthetases), STAT_1_ or Mx (myxovirus-resistance) that eventually cooperate to mount an antiviral state that restricts viral infection [20,23]. 

The potent antiviral action and pressure exerted by type-I IFNs on cells drives an evolutionary arms race between viruses and hosts leading to the perpetual selection of variants (natural mutant viruses) endowed with new polypeptide sequences that confer functions and activities that among others block IFN production, release and/or signaling thereby mediating evasion from host defense mechanisms. Thus, all naturally occurring viral pathogens express proteins that have acquired, upon accumulation of mutations, various properties and functions allowing them to block the innate immune response in their host [24,25]. While the latter has not yet been proved for rodent protoparvoviruses (PVs) (and for parvoviruses in general), it is well known and described that viruses belonging to other virus families (influenza virus, herpes simplex virus, dengue virus, SARS-CoV-2, etc.) are all expressing polypeptides that exert evasion functions including the blocking of type-I IFN production and/or signaling [26,27,28].

Owing to IFNAR ubiquitousness, IFN stimulation may also affect, besides cells adjacent and identical to the ones infected, immune cells belonging to the innate (NK and NKT cells, macrophages) and the adaptive immune system (DCs, T lymphocytes, B cells), as observed for instance in some tumor microenvironments (TMEs). Altogether, type-I IFNs contribute, as already mentioned for PAMPs and DAMPs, to the development of an efficient cellular antiviral immune response [20]. 

Notably, when the virus-targeted cell is malignant, as it is in the case of OVs, tumor antigens (TAs) may also be released/exposed upon cell death, representing an additional type of immunogenic factor being produced besides PAMPs, DAMPs and IFNs. Thereby, the cocktail of immunogenic molecules produced upon virus infection will acquire cumulative immunostimulating capacities. Thus, OV-triggered tumor cell death is a potentially immunogenic mechanism (immunogenic cell death (ICD)) that is also able to trigger, in addition to antiviral immunity, an anticancer immune response, the most valuable outcome of a successful immunotherapeutic treatment [6,29,30]. For example, the induction of type-I IFN expression in the TME was suggested to be categorized as an immunotherapy (including with checkpoint inhibitors) objective, as it is considered a good prognostic marker for the development of an efficient anticancer immune response [31,32,33]. 

Rodent protoparvoviruses (PVs) minute virus of mice, prototype strain (MVMp) and the rat H-1 parvovirus (H-1PV) are members of the *Parvoviridae* family and display oncolytic virus properties [1,34,35]. They are among the smallest viruses known, with a diameter of around 25 nm. They are composed of a linear single-stranded DNA (ssDNA) genome of ~5.1 kb encoding two capsid proteins (VP1 and VP2) and three non-structural polypeptides (NS1, NS2 and SAT). The expression of the NS genes is controlled by the early P4 promoter, while the late P38 promoter (*trans*-activated by NS1) regulates the expression of VP1, VP2 and SAT genes. Both MVMp and H-1PV demonstrated oncotropic and oncolytic properties in various preclinical in vitro and in vivo tumor models [1,35,36]. Furthermore, H-1PV safety, tolerability and immunogenic activity were shown in two clinical trials in glioblastoma (GBM) and pancreatic ductal adenocarcinoma (PDAC) patients [37,38]. Both trials demonstrated that also in human patients the virus was capable of inducing virus (tumor)-specific cellular immune responses leading to tumor infiltration with cytotoxic T lymphocytes, suggesting the establishment of an immunogenic intratumoral milieu. While the median progression-free and overall survival of the GBM patients was extended in comparison to recent meta-analyses, no complete cure was achieved in this first phase I/IIa trial, suggesting that both the oncolytic and the immunostimulating arms of H-1PV infection may be subjected to further improvement [39]. 

Given the beneficial effects of type-I IFNs on the induction of anticancer immune responses, the limited capacity of rodent PV to induce such responses may be explained by the inability of MVMp and H-1PV to induce type-I IFN production upon infection of transformed/tumor cells. In contrast, normal cells (e.g., mouse embryonic fibroblasts [MEFs], human peripheral blood mononuclear cells [hPBMCs]) release these cytokines upon PV infection [40,41]. While in MEFs the PRR, PAMP and downstream immune pathways have not yet been identified, IFN production induced in PV-infected hPBMCs could be attributed to the PRR TLR-9. Moreover, since in the latter cells neither replication nor transcription of viral genes or transduction of PV proteins could be detected, we hypothesized that the PAMP engaging TLR-9 is most likely (part of) the viral ssDNA genome, which comprises few potential and no typical activating CpG motifs [42]. Since MVMp was shown to replicate and transduce its proteins in infected MEFs while triggering a weak but nevertheless significant IFN production [40], we assume that other PRRs, apart from TLR-9, are likely to be responsible for this production and that the PAMP involved is other than the virus genome. 

Taking into account the relevance of type-I IFN production for the activation of an efficient anticancer response by immunotherapies, we set ourselves the goal of determining the nature of (i) the PAMP initiating IFN production in MVMp-infected MEFs and (ii) the PRR engaged. Moreover, since IFN production could never be detected in PV-infected transformed or tumor cells, a feature that may negatively impact PV ability to trigger potent and efficient anticancer immune responses, we sought to answer the question of whether any innate immune pathway is triggered by PV infections in these types of cells. Last but not least, we also investigated whether the lack of IFN production observed in transformed/tumor cells upon PV infections may be related to a PV-triggered evasion mechanism exerted specifically or most efficiently in neoplastic cells.

## 2. Materials and Methods

### 2.1. Antibodies, Kits and Compounds

The rabbit antiserum αSP8 and the monoclonal antibody 3D9 both raised against the parvoviral NS1 protein, as well as the rabbit polyclonal antibody SP6 raised against the parvoviral NS2 proteins, were described previously [43]. The polyclonal rabbit antiserum TATT3 raised against the capsid VP1 and VP2/VP3 proteins of MVMp was a generous gift of P. Tattersall (Yale University, New Haven, CT, USA). The monoclonal mouse B7 antibody directed against MVM capsids was a generous gift of J. M. Almendral (Centro de Biología Molecular Severo Ochoa (CSIC-UAM), Universidad Autónoma de Madrid, 28049 Cantoblanco, Madrid, Spain) [44]. The goat polyclonal anti-GAPDH and the rabbit anti-STAT_1_ and STAT_2_, as well as the mouse monoclonal anti-PKR antibody, were from Santa Cruz Biotechnology (Heidelberg, Germany). The polyclonal rabbit antibody directed against the phospho(Tyr^701^)- α and β isoforms of STAT_1_ was obtained from Cell Signalling (Frankfurt, Germany). The polyclonal rabbit antibody specific for phospho(Tyr^689^)-STAT_2_ was from Millipore (Schwalbach/Ts, Germany). The mouse monoclonal antibody directed against Actin was from MP Biomedicals (Heidelberg, Germany). The goat polyclonal (C-20, sc-6216) antibody directed against Lamin B was obtained from Santa Cruz Biotechnology (Heidelberg, Germany). The rabbit polyclonal antibody directed against NFĸB p65 (C-20, sc-372) was from Santa Cruz Biotechnology (Heidelberg, Germany). The goat polyclonal antibody raised against IRF-3 (C-20, sc-15991) was from Santa Cruz Biotechnology (Heidelberg, Germany). The mouse monoclonal antibody K1 raised against dsRNA was obtained from SCICONS (Szirak, Hungary). The synthetic low molecular weight double-stranded RNA (dsRNA) poly(I:C) was from GE Healthcare Europe (Freiburg, Germany). For transfection, Lipofectamine 2000 from Invitrogen (Karlsruhe, Germany) was used. ELISA kits for the detection of mouse and human IFN-β were obtained from R&D Systems (Wiesbaden, Germany). The TLR-9 agonist ODN 2395 and antagonist (ODN 2088) were obtained from Invivogen (Toulouse, France).

### 2.2. Cell Culture

Mouse-transformed A9 fibroblasts and Simian virus 40 (SV40)-transformed human newborn kidney NB324K cells were maintained in Minimum Essential Medium (MEM) supplemented with 5% heat-inactivated fetal bovine serum (FBS), 2 mM L-glutamine, 100 µg/mL penicillin and 10 units/mL streptomycin. HEK293 as well as the human cervix carcinoma cell line HeLa and the mouse B16 melanoma subclone B78/H1 were grown in Dulbecco’s Modified Eagle’s Medium (DMEM) containing 10% FBS and appropriate antibiotics. Wild type as well as knock out [45] low passage (<5) primary mouse embryonic fibroblasts (MEFs) freshly isolated from 12.5 to 13.5 days post-conception embryos of C57BL/6 mice were cultured in DMEM containing 10% heat-inactivated FBS with antibiotics. Primary C57BL/6 TLR3^−/−^ MEFs were a generous gift of R.W. Finberg (Department of Medicine, UMass Medical School, Worcester, MA, USA). Immortalization of primary MEFs using the 3T3 protocol [46] was performed upon cultivation of C57BL/6 embryonic cells over 20 to 25 passages in 10 cm dishes with Dulbecco’s Modified Eagle’s Medium (DMEM) containing 10% FBS and appropriate antibiotics. Trypsinization of approximately 80% confluent cultures was performed each third day.

### 2.3. Production and Titration of Virus Stocks

Primary stocks of wild-type MVMp and H-1PV were produced by calcium phosphate transfection of HEK293T cells using the pdBMVp infectious molecular clone of MVMp and the pSR19 infectious clone of H-1PV, respectively, as previously described [41]. Cells were harvested 3 days post-transfection, and viruses were collected by repeated cycles of freezing and thawing in vTE (50 mM Tris-HCl [pH 8.3], 0.5 mM EDTA). Crude cell extracts were then used to once re-infect human NB324K cells for a single further amplification of the stock. After subjecting infected NB324K cells to another series of free-thaw cycles in vTE buffer, virus stocks were purified by non-ionic iodixanol gradient centrifugation [47]. Viral stocks were titrated by plaque assays on human NB324K cell monolayers infected with serial dilutions of virus and expressed as pfu/mL. Inactivation of MVMp particles by UV exposure was performed by exposing half of a stock of virus to a Stratagene UV cross linker at 500 J/m^2^. The Lentogenic NDV *Ulster 2C paramyxovirus* strain was propagated in embryonated chicken eggs, harvested from the allantoic fluid, purified by ultracentrifugation as described [48] and cryopreserved in aliquots at −80 °C (generous gift of R. Zawatzky). The NDV stock was quantified by a hemagglutination assay. One hemagglutination unit (HU) is defined as the smallest virus concentration leading to visible sheep erythrocyte agglutination. 

### 2.4. Cell Infection

Cell monolayers were infected with viruses at the MOI indicated in each figure using serum-free media. After 1 h, inoculum was discarded, and complete medium was added onto the cells. They were then harvested following infection at times indicated in each figure.

### 2.5. Cell Transfection

Transfections of mock- or virus-infected cells grown in 10 cm dishes (MEFs, 8 × 10^5^/dish; A9, 1 × 10^6^/dish) were carried out using Lipofectamine 2000 according to the manufacturer’s instructions. Cells were transiently transfected with synthetic dsRNA poly(I:C) at a final concentration of 2 µg/mL for the times indicated before being processed for further analysis. Cultures were also transfected with total RNAs (1 µg/mL) extracted from mock-treated or virus-infected cells before being processed for further analysis. Briefly, the total RNAs were extracted using the Trizol or the RNeasy procedures following the manufacturer’s instructions from two 10 cm dishes/condition of MEF (8 × 10^5^), A9 (1 × 10^6^), HEK293 (1 × 10^6^) or HEK293T (1 × 10^6^) cells that were mock-treated or infected at the MOI and for the time indicated in each figure legend. The total extracted RNAs were subjected, or not, before their transfection to DNase I (RQ1; Promega, Madison, WI, USA) for 25 min at 37 °C before inactivation of the enzyme at 65 °C for 10 min, following the manufacturer’s protocol. In some experiments, DNase I-exposed total RNAs were further treated for 1 h at room temperature with RNase A or RNase T1 to hydrolyze ssRNAs, or with RNAse V1 to degrade dsRNAs (Invitrogen, Karlsruhe, Germany). In some cases, DNase I-exposed total RNAs were further subjected to Proteinase K (Roche Diagnostics, Basel, Switzerland) digestion to eliminate proteins or to Shrimp Alkaline Phosphatase (SAP; New England BioLabs) for 60 min at 37 °C before reaction was stopped by heating at 75 °C for 20 min and at 65 °C for 15 min, respectively. 

### 2.6. Viral DNA Extraction and Southern Blot Analysis

Viral DNA intermediates were isolated using a modified Hirt extraction method, as previously described [49]. Briefly, medium from mock-treated or parvovirus-infected cultures was discarded at the time points indicated in figure legends, and cells were scraped in PBS and pelleted by centrifugation at 500× *g* for 5 min at room temperature (RT). Cell pellets were resuspended in a 1:1 mixture (*v/v*) of vTE buffer and 2× Hirt buffer (20 mM Tris [pH 7.4], 20 mM EDTA, 1.2% SDS), followed by proteinase K digestion (400 µg/mL) for 18 h at 46 °C. Cellular genomic DNA was sheared by five passages through 0.5 and then 0.4 mm needles. DNA samples (3 µg) were fractionated by electrophoresis on a 0.8% agarose gel. After denaturation, the DNA was immobilized onto a nylon Hybond N^+^ membrane (Amersham Biosciences, Freiburg, Germany). Viral DNA intermediates were detected, after denaturation and neutralization, by hybridization with a ^32^P-labeled DNA probe corresponding to the *Eco*RV (nt 385)-*Eco*RI (nt 1084) fragment of the MVMp NS genes.

### 2.7. SDS-PAGE and Western Blot Analysis

At the indicated time points, mock-treated, transfected and/or infected cells were scraped in PBS and centrifuged at 500× *g* for 5 min at room temperature. Cell pellets were resuspended in a modified radio-immunoprecipitation assay (RIPA) buffer (50 mM Tris-HCl [pH 7.4], 150 mM NaCl, 1 mM EDTA, 1% NP-40, 0.25% Na-deoxycholate, protease inhibitor cocktail [Roche Diagnostics] and phosphatase inhibitors: 20 mM NaF, 5 mM *ß*-glycophosphate, 5 mM *p*-nitrophenyl phosphate, 5 mM sodium molybdate, 1 mM sodium orthovanadate, 5 mM sodium phosphate) and stored on ice for 30 min. Samples were centrifuged at 20,000× *g* for 15 min at 4 °C, and the protein concentration in the supernatants was determined using the Pierce BCA protein assay kit according to the manufacturer’s instructions (Pierce Biotechnology, Rockford, IL, USA). Samples were then boiled for 5 min in Laemmli buffer, fractionated by 8 or 10% SDS-PAGE and blotted onto nitrocellulose membranes (Schleicher & Schuell, Dassel, Germany). The membranes were then blocked with 1 X PBS containing 5% low-fat dry milk and 0.1% Tween-20 for 1 h. For the detection of phosphorylated proteins, 1× Tris-buffered saline solution (TBS: 20 mM Tris-HCl [pH 7.6], 137 mM NaCl) containing 0.1% Tween-20 and 2% casein was used as a blocking solution. Incubations with primary antibodies were carried out at 4 °C overnight either in 1 X PBS containing 5% low-fat dry milk and 0.1% Tween-20, or in 1× TBS supplemented with 0.1% Tween-20 and 5% bovine serum albumin. Individual proteins were identified by means of specific antibodies used at a 1:2000 (SP8, TATT3), 1:10,000 (Actin) or 1:1000 (others) dilution. Protein–antibody complexes were then visualized with horseradish peroxidase-conjugated anti-rabbit (1:10,000 dilution) or anti-mouse (1:5000 dilution) IgGs (Promega). The immunoreactive total and phosphorylated proteins were detected by ECL (Perkin Elmer Life Sciences) (Amersham Biosciences, Freiburg, Germany).

### 2.8. Fluorescence Microscopy

Cells were seeded on spot slides (3000 cells/spot) in 50 µL of complete medium. After 24 h, the medium was removed, and cells were mock-treated or infected for 1 h at 37 °C at the indicated MOI (50 µL inoculum in serum-free MEM). The inoculum was then removed and replaced with 100 µL of fresh MEM supplemented with 5% FBS. At indicated time points, cells were fixed in PBS containing 4% paraformaldehyde for 30 min and subsequently permeabilized in PBS containing 0.5% Triton X-100 for 10 min. Before staining, cells were incubated for 1 h at 37 °C in PBS containing 5% FBS as blocking solution. After extensive washing with PBS, cells were further incubated for 2 h at 37 °C in a PBS solution containing a 1:100 or a 1:1000 dilution of the anti-NS1 antibody 3D9 or SP8, respectively, a 1:50 dilution of the anti-NFĸB p65 and anti-IRF-3 antibodies and a 1:200 dilution of the anti-dsRNA K1 antibody. After being extensively washed in PBS, the preparations were incubated for 1 h at 37 °C with PBS containing a 1:600 dilution of secondary donkey anti-mouse, anti-rabbit or anti-goat IgGs conjugated to Alexa Fluor^®^ 594 or Alexa Fluor^®^ 488 (Molecular Probes, Eugene, OR, USA). Before mounting with Elvanol, the stained cells were incubated for 2 min with Hoechst solution to visualize the cell nucleus through DNA labeling and then extensively washed with PBS. Pictures were taken using an Olympus FluoView (FV1000) confocal microscope and the Olympus FV10-ASW (version 02.01) software. For some experiments, pictures were taken with a conventional epifluorescence microscope (Leica DMRBE; ×63 objective with immersion oil). Images were then captured using a Hamamatsu Orca digital camera and processed using Openlab 2 (Improvision, Bathurst, Australia). The analysis of colocalization was carried out using the ImageJ plugin RGB profiler (https://imagej.nih.gov/ij/plugins/rgb-profiler.html (first time accessed on 10 February 2010)) created by Christophe Laummonerie and Jerome Mutterer.

### 2.9. Detection of Type-I IFN Production

The secretion of type-I IFNs in the culture medium of virus-infected or RNA-transfected cell cultures was determined by enzyme-linked immunosorbent assay (ELISA). Briefly, culture supernatants of mock-treated or stimulated cells were harvested at indicated time points and cleared of cell debris by a brief centrifugation (500× *g* for 5 min). The concentrations of type-I IFN-β were then determined in the supernatants using PBL kits from R&D Systems (Wiesbaden, Germany) following the manufacturer’s instructions.

### 2.10. Statistical Analysis

Microsoft Excel 2016 was used to perform a paired two-tailed Student’s *t* test. Results were shown as the mean values of triplicates ± standard deviation of a representative experiment or as an average of at least two replicated independent experiments.

## 3. Results

We previously demonstrated that MVMp triggers a type-I interferon (IFN) production in primary MEFs (semi-permissive, i.e., undergoing MVMp replication to some extent but failing to release detectable amounts of progeny virions) but not in the established mouse fibroblast line A9 (fully permissive for MVMp infection) [40].

### 3.1. Parvovirus Infection Is Sensed by Both Normal and Transformed Cells but Results in Type-I IFN Production Only in the Former Cells

**Figure 1:**
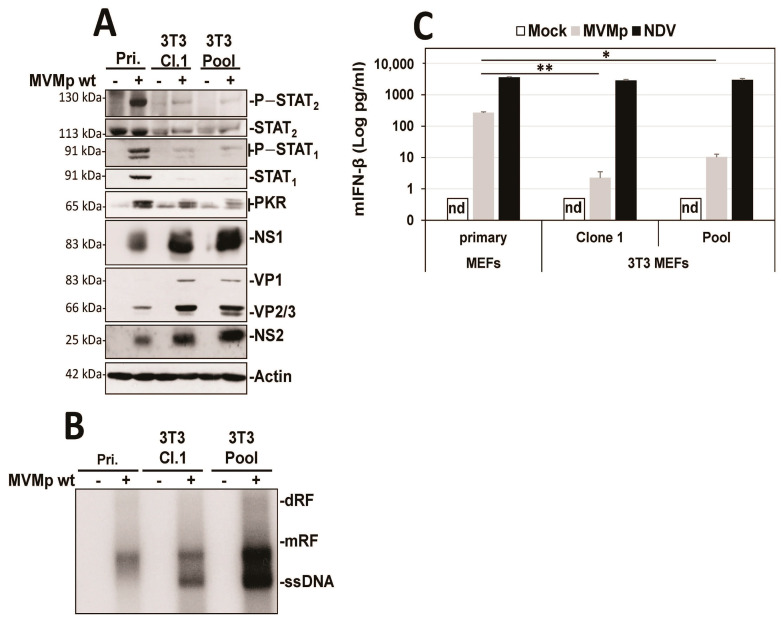
3T3 immortalization of primary MEFs is sufficient to prevent the development of an innate antiviral response upon MVMp infection. Primary MEFs (Pri.) and 3T3-immortalized mouse embryonic fibroblasts (Cl.1, clone 1; Pool, pool of 5 clones) were either mock-treated or infected with MVMp (MOI 5 pfu/cell) for 48 h. Samples were analyzed by (**A**) Western blot experiments using the indicated antibodies or by (**B**) Southern blot experiments using a ^32^P-labeled DNA probe specific for the NS1-encoding region of MVMp DNA: dRF, dimeric replicative form; mRF, monomeric replicative form; ssDNA, single-stranded genome. (**C**) Primary and 3T3-immortalized mouse embryonic fibroblasts (MEFs) were infected with (**C**) MVMp (MOI 5 pfu/cell) for 48 h or NDV (Ulster strain, 40 HAU/10^6^ cells) for 16 h. Culture supernatants were analyzed by ELISA to measure IFN-β concentration. Data are presented as mean + SD of three independent experiments. nd: not detected. Statistical significance was calculated using a paired two-tailed *t* test: * *p* < 0.05; ** *p* < 0.01.

Since the established mouse fibroblast cell line A9 is deficient in IFN induction upon MVM infection, we first tested whether this defect is a general property acquired during the cell immortalization process. To investigate this question, we used primary C57BL/6 MEFs and immortalized them using the 3T3 protocol [46]. In MVMp-infected immortalized 3T3 MEFs (Clone 1 or a pool of five different clones), we observed that the antiviral response induced (i.e., phosphorylation of STATs, induction of ISGs) was significantly reduced compared to that triggered in infected normal MEFs (Figure 1A). This reduction was moreover associated with a significant improvement in the ability of MVMp to translate its proteins (Figure 1A) and to replicate (Figure 1B) (increased presence of dRF (dimer replicative form), mRF (monomeric replicative form) and ssDNA (single-stranded genome in Southern blot experiments)) in 3T3 MEFs compared to primary MEFs. Similarly, the production and release of IFN-β upon infection was also massively reduced in both immortalized models relative to primary MEFs (Figure 1C); an effect not observed when these cells were infected with the potent stimulator of an antiviral innate immune response in mammalian cells [50,51], Newcastle disease virus (NDV Ulster strain) for 16 h. Altogether, our data indicate that the immortalization/transformation of semi-permissive cells may be associated with a loss of their ability to mount an antiviral innate immune response, hence the acquisition of increased permissiveness to parvovirus infection. 

**Figure 2:**
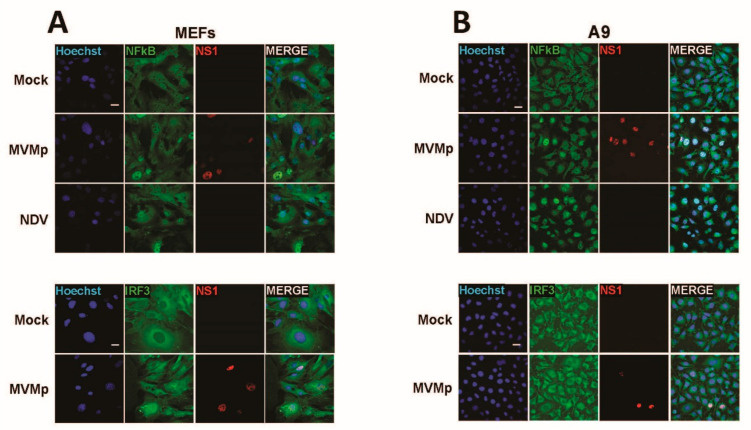
MVMp infection of permissive primary and transformed mouse fibroblasts induces nuclear translocation and thereby activation of NFĸB and IRF3 transcription factors. (**A**) Primary (MEFs) and (**B**) transformed (A9) mouse fibroblasts were either mock-treated or infected with MVMp (MOI 2 pfu/cell) for 48 h or with NDV (Ulster strain, 40 HAU/10^6^ cells) for 16 h. NS1 (red), NFĸB (green) and IRF3 (green) expression and subcellular distribution were analyzed using indirect immunofluorescence. Nuclei were visualized using Hoechst staining. Scale bar: 20 µm (**A**,**B**).

Our latter observations as well as the known essential contribution of NFĸB and IRF3 transcription factors in the development of type-I IFN response upon activation of an upstream PRR in infected cells brought us to investigate whether a difference in activation of both factors could be detected upon parvovirus infection in primary vs. transformed/tumor cells. Thus, we analyzed by indirect immunofluorescence the distribution of both transcription factors in primary MEFs as well as in transformed mouse fibroblasts (A9 cell line) known to be most permissive to MVMp infection and unable to induce any IFN-β production upon infections [40]. It has to be mentioned, however, that transfection of A9 fibroblasts with the artificial dsRNA poly(I:C) [40] or infection with NDV (Figure S1) are both triggering production of this cytokine, indicating that these transformed cells are intrinsically endowed with some functional PRR pathways. As expected, similar to NDV-infected cells (16 h p.i.), primary MEFs infected for 48 h with MVMp showed obvious nuclear translocation of both NFĸB and IRF3 (Figure 2A) compared to mock-treated cells, indicating that an innate antiviral pathway was indeed triggered by MVMp. To our surprise, similar observations were also made in MVMp-infected A9 fibroblasts 48 h p.i. (Figure 2B), although no IFN-β transcription, production nor release could ever be detected in such MVMp-infected cells. B78 mouse melanoma cells showed similar results (Figure S2).

**Figure 3:**
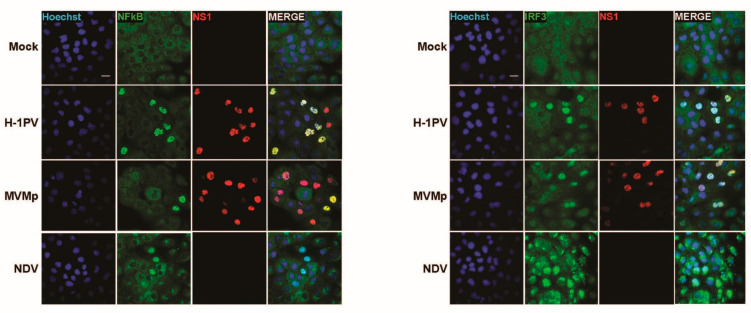
Parvovirus infection of permissive SV40-transformed human newborn kidney (NB324K) cells induces nuclear translocation and thereby activation of NFĸB and IRF3 transcription factors. NB324K cells were either mock-treated or infected with MVMp (MOI 2 pfu/cell) or H-1PV (2 pfu/cell) for 36 h. Processed samples were analyzed by indirect immunofluorescence using NS1- (red), NFĸB- (green) or IRF3- (green) specific antibodies. Nuclei were visualized using Hoechst staining. Scale bar: 20 µm.

To further validate these observations, we undertook similar experiments in the human SV40-transformed cell line NB324K that is highly permissive to both rodent parvoviruses MVMp and H-1PV (rat). These cells were previously shown to be endowed with functional type-I IFN-producing pathways, in particular upon poly(I:C) transfection or NDV infection, but they failed to produce type-I IFNs upon PV infection [41]. Here too, we observed in a number of infected cells (i.e., showing nuclear NS1 expression) a concomitant nuclear expression of NFĸB and IRF3 (Figure 3). Similar observations were made when NB324K cells were infected with a higher MOI (20 pfu/cell) (Figure S3A) or in human Hela cells upon MVMp infection (Figure S3B). Altogether, our data clearly indicate that during a productive parvovirus infection both transcription factors, NFĸB and IRF3, translocate into the nucleus of infected cells and activate an upstream PRR-dependent pathway. Since some infected cells, in particular transformed ones, do not show such effects although their nucleus is filled with NS1, it could be anticipated that the viral signals sensed by the PRR machinery may be transiently expressed and occur only at a certain time point during the parvovirus life cycle.

### 3.2. Parvovirus Sensing Can Take Place Independently of Conventional TLR and RLR Receptors and Requires Some Extent of Parvovirus Replication

**Figure 4:**
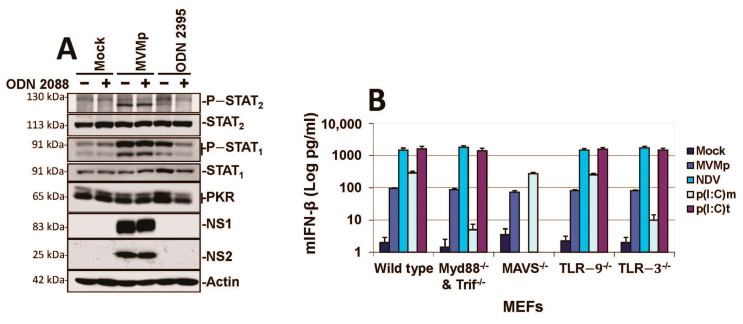
MVMp infections are not sensed by TLRs nor RLRs in primary mouse fibroblasts. (**A**) Primary wild-type C57BL/6 MEFs were pretreated or not with 10 µM of the TLR-9 inhibitor ODN 2088, then either mock-treated, MVMp-infected (MOI 10 pfu/cell) or stimulated with 4 µM ODN 2395 (a TLR-9 agonist), before being processed for Western blotting using the indicated antibodies. (**B**) Wild-type, Myd88/TRIF^−/−^, MAVS^−/−^ and TLR-9^−/−^and TLR-3^−/−^ primary MEFs were either mock-treated, MVMp-infected for 48 h, NDV-infected for 16 h, poly(I:C)-transfected (p(I:C)t, 2 µg/mL) or poly(I:C)-treated (p(I:C)m, 50 µg/mL). IFN-β levels were determined in the cell-free culture medium. Data are gathered from three independent experiments performed in triplicate and presented as mean ± SD.

**Figure 5:**
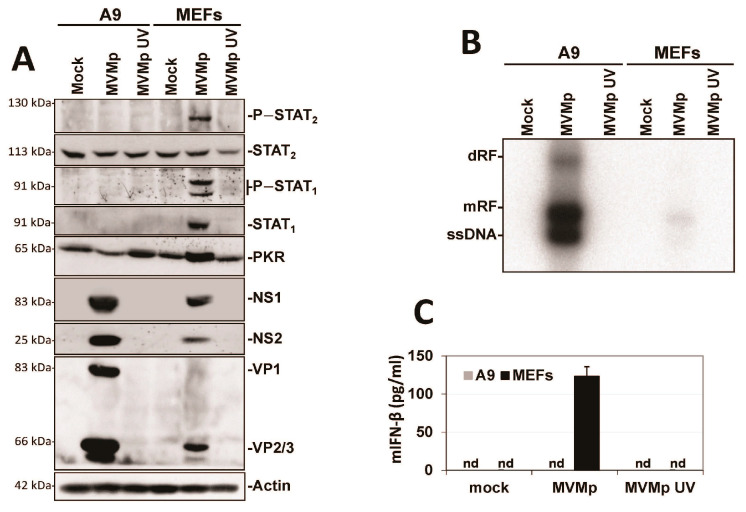
Protoparvovirus replication is required for the activation of an antiviral innate immune response in infected primary MEFs. Transformed (A9) and primary (MEFs) mouse fibroblasts were mock-treated or infected with equal doses of either wild-type or UV-inactivated MVMp corresponding, for the former stock, to an MOI of 5 pfu/cell. (**A**) Samples were collected 24 h post treatment and analyzed by Western blot for the activation of a type-I IFN-dependent antiviral response (based on STAT_1_/STAT_2_ transcription factor phosphorylation) and for the induction of ISG (PKR, STAT_1_ and STAT_2_) expression. Each blot is representative of three experiments that produced similar results. (**B**) Samples were processed for Southern blot analysis. The expression of viral DNA intermediates, namely, dRF (dimeric replicative form), mRF (monomeric replicative form) and ssDNA (single-stranded DNA genome) was investigated using a ^32^P-labeled DNA probe corresponding to a specific fragment of the viral NS-coding genomic region. The illustrated blot is representative of three experiments that gave similar results. (**C**) IFN-β production was quantified in the cell-free culture medium from mock-, MVMp wild-type- and MVMp UV-infected A9 and MEF cultures using ELISA and based on standard curves generated with recombinant IFN-β. Data are presented as mean ± SD from three experiments performed in triplicate.

Since TLR-9 is the PRR shown to sense rodent protoparvovirus infections (MVMp and H-1PV) in hPBMCs [41]), we next assessed whether this sensor is also involved in the innate response triggered by replicating MVMp in MEFs. We first observed that the TLR-9 inhibitor ODN 2088 was unable to prevent the phosphorylation of STAT_1_ and STAT_2_ transcription factors triggered by MVMp infections in primary MEFs (Figure 4A). This inhibitor was, however, able to block a weak stimulation of these immune factors by a TLR-9 agonist (ODN 2395). Moreover, MEFs isolated from wild-type, TLR-9^−/−^, TLR-3^−/−^, MAVS^−/−^ and MyD88^−/−^ and TRIF^−/−^ C57BL/6 mice embryos were all producing similar levels of IFN-β upon MVMp infection (Figure 4B), indicating that neither TLRs nor RLRs are involved in the IFN response triggered by replicating MVMp in MEFs. Based on these data, we can assume that TLR-9 is unlikely to be the PRR that triggers a type-I IFN response upon MVMp infection of MEFs. It should, however, be stated that TLR-9 agonists ODN 2395 (Figure 4A) and ODN 1826 (data not shown) trigger only a weak IFN response in primary MEFs (i.e., only slight phosphorylation of STAT_1_ and even no phosphorylation of STAT_2_). Accordingly, these antagonists failed to induce detectable IFN-β production in these cells, indicating that MEFs may harbor an intrinsic deficiency in their TLR-9 pathway, making them an inappropriate model for investigating the TLR-9 contribution to the antiviral response induced by replicating MVMp. We then tested whether protoparvovirus replication is required for type-I IFN synthesis in infected primary MEFs. We therefore analyzed by Western blot and IFN-β ELISA experiments whether UV-inactivated MVMp virions generate, similar to their non-irradiated counterparts, an IFN-dependent antiviral response in these cells. The UV inactivation of MVMp was confirmed by showing the inability of irradiated virus to induce at 40 h p.i. a nuclear expression of NS1 proteins in A9 cells (Figure S4A). We observed that only non-irradiated, replication-competent MVMp induces hallmarks of an antiviral innate response (i.e., phosphorylation of STAT_1_ and STAT_2_ transcription factors, induction of the expression of ISGs STAT_1_, STAT_2_ and PKR, as well as IFN-β production) in infected MEFs. These effects are not observed in MEFs infected with UV-inactivated particles (Figure 5A–C) nor in A9 cells subjected to active or UV-inactivated MVMp infections. This absence of IFN production upon UV-inactivated MVMp infection is not related to an alteration in the uptake of UV-treated particles since both wild-type and UV-exposed virions can be detected 12 h p.i. in the cytoplasm of highly permissive mouse A9 fibroblasts in the vicinity of the nucleus by immunofluorescence using an MVMp capsid antibody (Figure S4B). Altogether, these experiments indicate that replication of MVMp is required for the activation of a type-I IFN-dependent antiviral response in infected MEFs.

### 3.3. Parvovirus-Induced dsRNA Is a PAMP Candidate for PV Sensing

**Figure 6:**
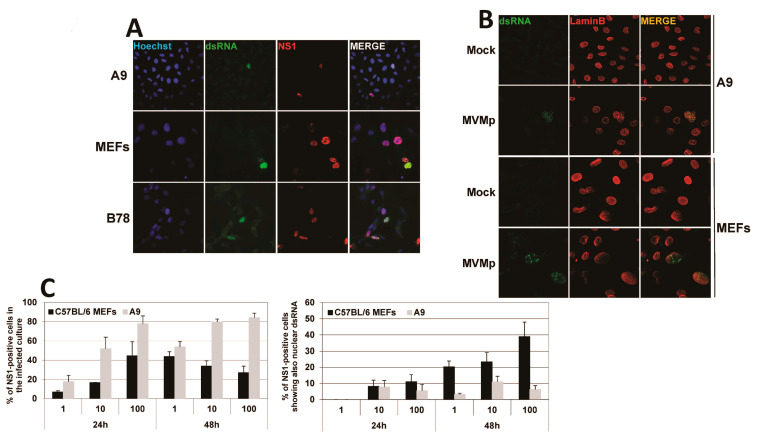
Nuclear dsRNA expression is detected in parvovirus-infected cells. (**A**) Primary MEFs, A9-transformed mouse fibroblasts and B78 mouse melanoma cells were infected with MVMp at, respectively, 10, 10 and 20 pfu/cell for 30 h and processed for indirect immunofluorescence using dsRNA- (green) and NS1- (red) specific antibodies. Nuclei were labeled using Hoechst staining. (**B**) MVMp-infected primary MEFs and A9-transformed mouse fibroblasts were analyzed by indirect immunofluorescence using dsRNA- (green) and lamin B- (red) specific antibodies. (**C**) Primary MEFs and transformed mouse A9 fibroblasts were infected with MVMp and analyzed by indirect immunofluorescence using dsRNA- and NS1-specific antibodies. For each condition, ten fields with at least 50 cells/field were counted, and the percentage of NS1- and dsRNA-positive cells was calculated. Data are presented as mean + SD from three independent experiments.

We next investigated which viral factor could trigger PRR pathways in (semi-)permissive parvovirus-infected cells. Given that viral nucleic acids (DNA and/or RNA) and in particular viral dsRNA are key pathogen-associated molecular patterns (PAMPs) activating PRRs upon infection with various viruses, we investigated by indirect immunofluorescence, using the mouse monoclonal K1 antibody directed against dsRNA, the presence of such a type of nucleic acid in MVMp-infected cells. As shown in Figure 6A and Figure S5A, dsRNA could be detected in MVMp-infected (i.e., NS1-positive) MEFs, A9 and B78 cells, but not in non-infected or mock-treated cells (Figure 6B and Figure S5A). To our surprise and in contrast with many viruses, which express such molecules in the host cell cytoplasm, we noticed that parvovirus infections lead to an almost exclusively nuclear accumulation of dsRNA. This dsRNA production correlated with PV replication as apparent from its overlap with NS1 expression (Figure 6A and Figure S5A). Indeed, the presence of dsRNA in infected cultures of MEFs or A9 cells is almost exclusively (i.e., ≥99% of dsRNA-positive cells) observed within the Lamin B-labeled nucleus compartment (Figure 6B and Figure S5B). We further noticed that the pattern of expression of dsRNA upon K1 labeling in parvovirus-infected cells consists most often in a nuclear dot-like motif that can in some rare cases completely fill the nucleus (Figure 6A and Figure S5A,B). This observation contrasts strikingly with the pattern observed upon transfection of both cell types with synthetic dsRNA poly(I:C) that also shows a dot-like motif but is, however, located into the cytoplasm of transfected cells (Figure S5C). Interestingly, only a fraction of infected (i.e., NS1-positive) MEFs, A9 or B78 cells show nuclear dsRNA expression at the time of harvesting (Figure 6C and Figure S5A). Of note, while the amount of dsRNA-positive A9 cells did not show a clear time- and MOI-dependence, an increase in time and MOI resulted in a higher percentage of infected MEFs showing nuclear dsRNA (Figure 6C). A concomitant nuclear expression of dsRNA and NS1 was also observed in MVMp-infected B78 mouse melanoma cultures (Figure S5D), as well as in transformed human newborn kidney (NB324K) and embryonic kidney 293 (HEK293) cells (Figure S6A–C). In both latter human cell types, this effect was observed upon MVMp and H-1PV infection, a feature reflecting the well-known high permissiveness of these cell lines to both parvoviruses (Figure S6A–C). These observations may be put together with the previously mentioned nuclear translocation of NFĸB and IRF3 in parvovirus-infected NB324K cells (Figure S3 and Figure 3).

**Figure 7:**
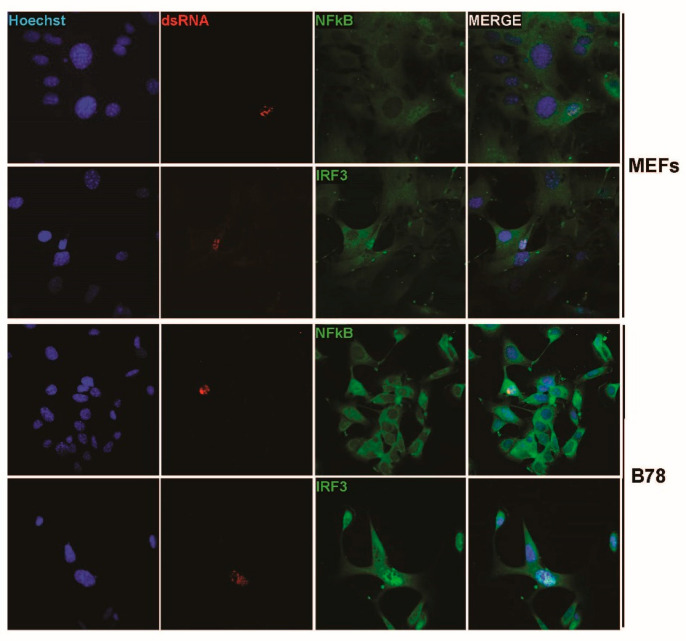
MVMp-infected MEFs and B78 cells show nuclear (co)localization between dsRNA and either IRF3 or NFκB transcription factors. Primary MEFs and B78 mouse melanoma cells were infected with MVMp and analyzed by indirect immunofluorescence using dsRNA- (red) and either NFĸB- (green) or IRF3- (green) specific antibodies.

Most importantly, IRF3 and NFκB translocation in MVMp-infected MEF or B78 cells was specifically detected in nuclei, which concomitantly expressed dsRNA. This colocalization suggests that the presence of the nucleic acid moiety in parvovirus-infected cells is directly or indirectly associated with the triggering of a PRR-dependent response (Figure 7). Altogether, our data indicate that MVMp and H-1PV infections lead to the nuclear expression of potentially immunogenic molecules, dsRNAs, usually considered as potent PAMPs in infected cells.

**Figure 8:**
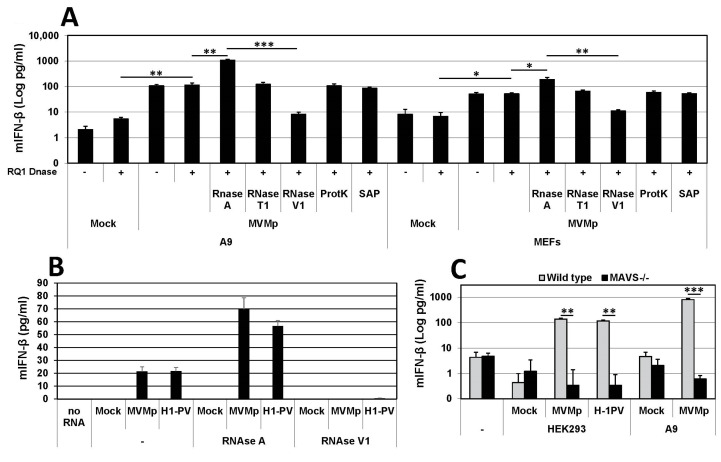
Double-stranded RNAs extracted from parvovirus-infected cells induce a MAVS-dependent IFN response upon transfection in MEFs. (**A**) Primary wild-type MEFs were transfected with 1 µg/mL total RNA extracted 48 h post parvovirus infection of transformed mouse A9 fibroblasts or primary MEFs. Total RNAs were further subjected, or not, to other treatments, as indicated in Materials and Methods. IFN-β release was measured 24 h after transfection by ELISA. Statistical significance was calculated using a paired two-tailed *t* test: * *p* < 0.05; ** *p* < 0.01; *** *p* < 0.001. Error bars for all data indicate the mean values ± SD of four independent experiments performed in triplicate. (**B**) Primary wild-type MEFs were transfected with 1 µg/mL total RNA extracted 24 h post mock-treatment, MVMp (MOI of 5 pfu/cell) or H-1PV (MOI of 10 pfu/cell) infection of HEK293 human embryonic kidney cells. Total RNAs were further subjected, or not, to other treatments, as indicated in Materials and Methods. IFN-β release was measured by ELISA 24 h after transfection. Data are presented as mean + SD of three independent experiments performed in duplicate. (**C**) Primary wild-type and MAVS^−/−^ MEFs were transfected with total RNA which was either extracted 48 h p.i. from A9 cultures infected, or not (Mock), with MVMp (MOI 1 pfu/cell) or isolated 36 h p.i. from HEK293 monolayers infected, or not (Mock), with MVMp (MOI 5 pfu/cell) or H-1PV (MOI 10 pfu/cell). Quantification of IFN-β released into the cell-free medium was performed by ELISA. Data are presented as mean + SD of three independent experiments performed in duplicate. Statistical significance was calculated using a paired two-tailed *t* test: ** *p* < 0.01; *** *p* < 0.001.

In order to characterize in more detail the nature as well as the PAMP behavior of the dsRNA expressed in parvovirus-infected cells, we extracted 48 h p.i. the total RNAs from mock-treated or parvovirus-infected cells using Trizol extraction and transfected naïve primary mouse fibroblasts (MEFs) with 1 µg/mL of these RNAs using Lipofectamine 2000. The measurement of IFN-β production in the cell-free medium of transfected MEFs was performed 24 h later by ELISA. Before being transfected, the total extracted RNAs were treated, or not, with DNase (RQ1, Promega) to destroy any potential DNA contamination. They were then treated, or not, either by RNase A to hydrolyze single-stranded (ss) RNAs such as mRNA at C and U residues, by RNase T1 that degrades ssRNAs at G residues or with RNase V1 that hydrolyzes double-stranded (ds)RNAs and cleaves substrates of at least six stacked nucleotides in a sequence-unspecific manner. Some DNase-treated total RNAs were also subjected to proteinase K digestions to eliminate any contaminating protein from the total extracted RNAs or to shrimp alkaline phosphatase (SAP) to dephosphorylate dsRNAs at their 5′ end, thereby inactivating for instance the main RIG-I ligands, 5’-PPP-dsRNAs [52,53]. As presented in Figure 8A, we indeed observed the production and release of IFN-β in the culture medium of MEFs only when these cells were transfected with the total RNA extracted from MVMp-infected A9 or MEF cells and never upon transfection with RNAs isolated from mock-treated cells. Of note, while DNase treatment had no significant impact on the response, RNase A treatment, but not RNase T1, induced a significant increase in the amount of cytokine produced. Moreover, when the total RNA previously digested with DNase was further treated with RNase V1, no IFN-β expression could be detected, suggesting that dsRNAs are indeed triggering this cytokine production (Figure 8A). We also noticed that the type-I IFN production was overall higher upon the transfection of RNAs isolated from infected A9 cells than from MEFs, although transfected RNAs originated from infections performed at a similar MOI and duration. Proteinase K or phosphatase (SAP) treatments had no effect on the IFN-β production induced by transfected RNAs. Taken together, these results indicate that dsRNAs produced in permissive parvovirus-infected cells are able to trigger an IFN response upon their artificial introduction into naïve cells. They also suggest that some ssRNAs or sequences thereof are either produced by parvovirus infections to limit or reduce the dsRNA-induced IFN-β production or that the hydrolysis of stretches of ssRNA sequence present between dsRNA motifs may increase the ability or amount of dsRNA molecules able to trigger cytokine production. Similar results were obtained when A9 cells were transfected instead of MEFs (data not shown) or when MEFs were treated with total RNAs extracted from MVMp or H-1PV-infected human HEK293 cells (Figure 8B).

Finally, since intracellular/cytosolic dsRNAs are usually sensed by cytoplasmic RLRs such as RIG-I or MDA-5 that both signal through the downstream adapter protein MAVS, we tested whether dsRNAs produced during a parvovirus infection harbor structural features allowing their recognition by these PRR pathways. We therefore compared the ability of wild-type and MAVS-deficient primary MEFs to trigger IFN-β expression upon transfection with DNase- and RNase A-treated total RNAs extracted from HEK293 or A9 cells that were either mock-treated or infected with MVMp or H-1PV. As shown in Figure 8C, we indeed detected type-I IFN production in culture media from wild-type MEFs stimulated with total RNA isolated from infected A9 or HEK293 cells but never in media from MAVS-deficient transfected MEFs. These data clearly indicate that dsRNAs produced by parvovirus infections have structural characteristics and properties allowing their recognition by an RLR/MAVS antiviral pathway that eventually leads to IFN-β production. However, such a situation is most likely artificial since it occurs because transfection allows the penetration of these dsRNA into the cell cytoplasm. Parvovirus infections normally do not show expression of dsRNAs in the cell cytoplasm in immunofluorescence experiments. However, as already mentioned, on very rare occasions, we observed in infected cells (i.e., less than 1% of dsRNA-positive A9 or MEFs) cytoplasmic localization of dsRNA (Figure S7A), however, always in association with the presence in this cellular compartment of the viral protein NS1 (Figure S7B,C). Taken together, these observations suggest that the cytoplasmic presence of dsRNA was most likely related to cytopathic effects triggered by the infection that led to a loss of integrity of the nuclear wall (Figure S7). The contribution of RLRs to the IFN-β production upon PV infection can nevertheless be ruled out so far since we have previously observed that MVMp-infected primary MEFs deficient for MAVS expression produce IFN-β to similar levels as wild-type cells (Figure 4). These data further account for the lack of contribution of this adapter protein and its upstream RLRs to the IFN response triggered by rodent parvovirus. It remains thus to determine whether PV-induced nuclear dsRNA can also act as a PAMP during an infection and contribute to IFN induction upon recognition by so far unknown nuclear PRRs.

It is important to note here that our findings contrast with previous observations showing that RNAs extracted from parvovirus-infected MEFs using the RNeasy kit from Qiagen do not trigger any antiviral response when transfected into TLR-7-deficient bone marrow cells [54]. This discrepancy led us to compare the IFN-β production ability of total RNAs isolated either using the Trizol procedure or following the RNeasy kit protocol. A comparison was made using the same initial batch of infected A9 cells that was subdivided into two equivalent fractions. Interestingly, we observed that while total RNAs extracted using the Trizol procedure were indeed able to trigger an IFN response when transfected into recipient primary wild-type MEFs, RNAs obtained using the RNeasy kit were unable to trigger such a response (Figure S8). Altogether, this experiment suggests that during the RNeasy kit isolation procedure, the parvovirus-produced RNAs get lost. Given that a threshold in the size of RNAs able to be retained on the RNeasy column is known (i.e., around 200 nucleotides), it is tempting to speculate that the size of the dsRNAs produced in parvovirus-infected cells is below 200 nucleotides. Thus, the small size of the (ds) ribonucleic acids produced in parvovirus-infected cells most likely accounts for the opposite results obtained between our present investigation and the one published previously.

**Figure 9:**
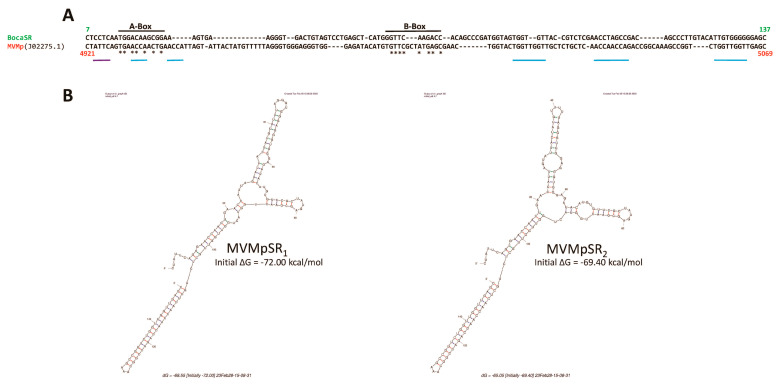
The 3′ extremity of the MVMp genome contains a region that shares sequence homology with BocaSR. (**A**) SnapGene 6.0.5-based alignment between the sequence of the BocaSR-transcribing region of HboV1 and the full MVMp genome (NCBI accession # J02275.1). Numbers indicate the position of nucleotides of each sequence that were aligning. A- and B-boxes of Pol III in the BocaSR sequence are indicated. Asterisks refer to identical nucleotides between the BocaSR and the MVMp DNA sequence within both Box domains. Blue lanes correspond to NS1 binding domains (ACCA/TGGT motif) within the MVMp sequence, while the purple lane corresponds to an NS1 nick site. (**B**) Predicted secondary structures of MVMpSR using the UNAFold algorithm [57]. For each structure, a thermodynamic value reflecting its potential stability is indicated.

**Figure 10:**
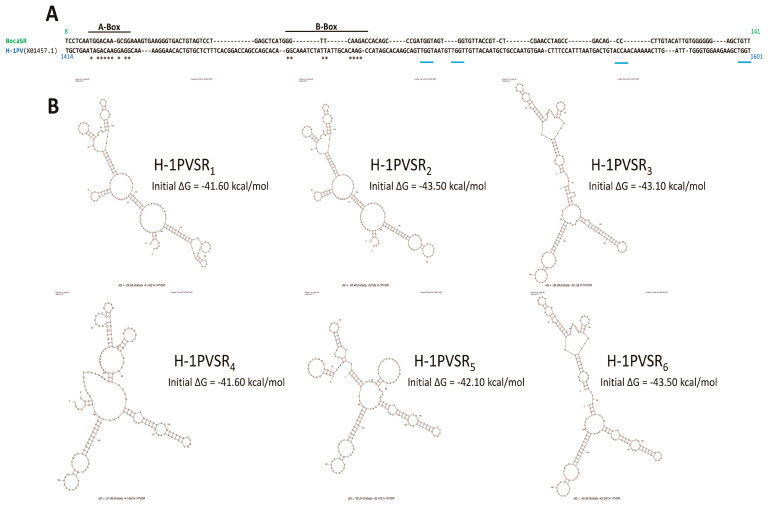
The H-1PV genome contains a region within its NS-coding genes that shares sequence homology with BocaSR. (**A**) SnapGene 6.0.5-based alignment between the sequence of the BocaSR-transcribing region of HboV1 and the full H-1PV genome (NCBI accession # X01457.1). Numbers indicate the position of nucleotides of each sequence that were aligning. A- and B-boxes of Pol III in the BocaSR sequence are indicated. Asterisks refer to identical nucleotides between the BocaSR and the H-1PV DNA sequence within both Box domains. Blue lanes correspond to NS1-binding domains (ACCA/TGGT motif) within the H-1PV sequence. (**B**) Predicted secondary structures of H-1PVSR using the UNAFold algorithm [57]. For each structure, a thermodynamic value reflecting its potential stability is indicated.

Interestingly, our observation of a nuclear dsRNA expression in parvovirus-infected cells is not without precedent. Indeed, Jianming Qiu’s group already observed such a feature while infecting human cells with another parvovirus belonging to the genus *Bocaparvovirus* [55], human bocavirus 1 (HBoV1) [56], an autonomously replicating parvovirus similar to MVMp and H-1PV but with distinct features [55]. Qiu’s study revealed that the HBoV1 genome contains in its 3′ noncoding region (nucleotides [nts] 5199 to 5338) a gene that encodes a noncoding, 140 nucleotide(nt)-long, dsRNA, termed BocaSR, that shares features with the small noncoding RNAs expressed by adenoviruses (VA RNAs) or the Epstein–Barr virus (EBERs). BocaSR localizes into the nucleus of infected host cells and contributes to HBoV1 replication. Based on these observations, we decided to align using the SnapGene 6.0.5 program, the BocaSR nt sequence with that of the whole MVMp (NCBI accession #: J02275.1) or H-1PV (NCBI accession #: X01457.1) genome. As presented in Figure 9A, we surprisingly observed that one sequence, located in the 5′ noncoding region of MVMp (nt 4921 to 5069), shares 51.8% identity with the BocaSR sequence. Moreover, using the UNAFold algorithm [57] (http://www.unafold.org/mfold/applications/rna-folding-form.php (First time accessed on 9 May 2022)), we obtained results indicating that this MVMp DNA sequence of 149 nts could indeed give rise to two secondary RNA structures closely mimicking those (two) proposed for BocaSR by the same program (Figure 9B and Figure S9). They were indeed made of several dsRNA stretches separated by loops and bubbles, as already proposed for BocaSR [56]. Noteworthy, both putative MVMp dsRNA structures seem to be more stable than those two proposed for HBoV1, with a ΔG of −72.0 or −69.4 kcal/mol for the former ones compared to only −56.3 or −58.8 kcal/mol for the latter ones; a feature that might be related, at least in part, to their slightly larger length. Regarding the H-1PV alignment, the results are less straightforward. Indeed, a similar approach as that used previously for MVMp actually revealed the presence of a sequence (nucleotides 1414 to 1601) sharing 48.7% sequence homology with BocaSR (Figure 10A). However, its localization into the NS gene unit (nts 264 to 2282) presently questions its existence. Moreover, analysis of this small H-1PV sequence (188 nts) using the UNAFold program led to a prediction of six different RNA structures composed of dsRNA stretches surrounded by bubbles and loops (Figure 10B). The stability of these putative H-1PV RNA structures seemed moreover weaker than those encoded by the MVMp or HBoV1 viruses, as demonstrated by ΔG values ranging from −41.6 to −43.5 kcal/mol.

Alternatively, when alignment was performed between the putative MVMp DNA sequence encoding a putative dsRNA molecule (MVMpSR) and the whole H-1PV genome, it revealed the presence of a DNA region located also in the 5′ noncoding region (nts 4932–5076, 145 nts) of the H-1PV genome sharing 84,6% sequence homology with that of MVMp (Figure S10A). Such a result is not surprising taking into account the high DNA sequence homology existing between both PV genomes. Analysis of the potential RNA structures being produced by this H-1PV DNA segment revealed in contrast to MVMp or HBoV1, up to nine different potential RNA structures, with ΔG ranging from −45.7 to −39.6 kcal/mol (Figure S10B). Some of them were indeed endowed with stability constants that were, on the one hand, higher than those proposed for the putative region localized within the NS unit gene of H-1PV but, on the other hand, weaker than those proposed for MVMp or HBoV1.

Altogether, our in silico analyses identified in MVMp and to a lesser extent in H-1PV, DNA regions located within the 5′ end of the respective genomes showing structural homologies with the BocaSR sequence. Unfortunately, while BocaSR was clearly shown to possess the promoter sequences (A-Box, B-Box and termination signal) required for polymerase III-dependent transcription, we did not find, apart from some strong homologies in the A-Box, such features in the small DNA sequences of protoparvoviruses MVMp or H-1PV. However, we noticed that several NS1-binding sites (ACCAACCA motif) and even an active NS1 nick site (ACTATTC) were present in the MVMp DNA region (Figure 9A and Figure S10A).

### 3.4. IFN Induction Aborts in Transformed PV-Infected Cells That Evade the Antiviral Response

The above results show that rodent parvovirus infections result in nuclear dsRNA accumulation of both normal (semi-permissive) and transformed (permissive) cells, associated with the nuclear translocation/activation of NFĸB and IRF3 transcription factors, but that IFN-β production is restricted to normal cells. These observations lead us to speculate, first, that parvoviruses are endowed with an evasion mechanism that blocks type-I IFN production in host cells and, second, that transformed/malignant cells offer a most suitable environment for the efficient completion of this evasion process at a stage following virus sensing and the activation of a PRR signaling. To test this hypothesis, we compared the abilities of MVMp pre-infected A9 and primary wild-type MEF cultures to trigger an IFN-β production upon their further challenge with well-known inducers of IFN production, in particular, synthetic dsRNA poly(I:C) transfection or paramyxovirus NDV infection.

**Figure 11:**
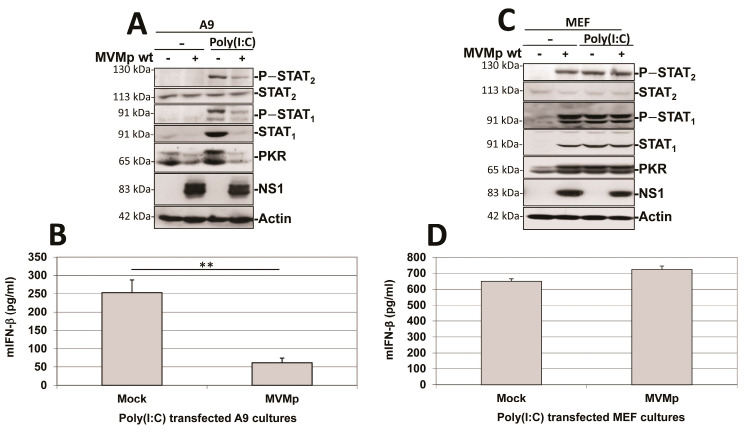
MVMp pre-infection of transformed (A9), but not of primary (MEF), mouse fibroblasts prevents further poly(I:C)-triggered IFN-β production. Cultures of transformed mouse A9 fibroblasts (**A**,**B**) or primary MEFs (**C**,**D**) were either mock-treated or MVMp-infected (MOI of 10 pfu/cell) for 24 h. Cultures were transfected, or not, with poly(I:C) and culture medium was replaced by fresh complete medium 5 h after transfection. Cells were further cultivated for 10 h before being processed for Western blotting (**A**,**C**) as described in Material and Methods. IFN-β was measured by ELISA (**B**,**D**) in culture supernatants. Data are presented as mean + SD of three independent experiments performed in triplicate. Statistical significance was calculated using a paired two-tailed *t* test: ** *p* < 0.01.

As shown in Figure 11A, pre-infection of transformed fibroblast (A9) cultures with MVMp strongly reduced the ability of secondary poly(I:C) transfection to activate the IFN-responsive pathway in these cells. Poly(I:C) transfection was able to trigger the IFN responsive pathway in mock-treated A9 cultures, as revealed by the phosphorylation of STAT_1_ and STAT_2_ transcription factors and the induction of the expression of ISGs, STAT_1_ and PKR. In contrast, these effects were strongly (STAT phosphorylation) or fully (ISG induction) prevented in cultures previously infected by the parvovirus. Accordingly, IFN-β release triggered by poly(I:C) transfection was strongly reduced when A9 monolayers were pre-infected with MVMp (Figure 11B). Similar observations were made regarding the ability of transfected poly(I:C) to induce IFN-β production in human transformed cell lines NB324K, Hela, HEK293 and HEK293T that were, or not, pre-infected with MVMp or H-1PV (Figure S11A). Indeed, pre-infection of all these permissive cultures with either parvovirus induced an important reduction in the IFN-β production triggered by transfected poly(I:C). These results show that the effects observed are not limited to MVMp nor to mouse fibroblasts and can be extended to transformed/tumor cells in general. It is important to note also that the parvovirus NS1 expression in infected A9 monolayers was not significantly impaired by the synthetic dsRNA transfection (Figure 11A), indicating the failure of the IFN inducer to interfere with parvovirus replication.

This PV-dependent inhibition of poly(I:C)-induced IFN production was not observed in MVMp-infected primary MEF cultures. Parvovirus infection by itself triggered an IFN response (i.e., phosphorylation of STAT_1_ and STAT_2_ transcription factors and induction of the expression of STAT_1_ and PKR associated with IFN-β production and release) in MEF cultures, as previously mentioned (Figure 5). MVMp pre-infection failed to reduce the IFN response taking place in dsRNA-transfected MEFs (Figure 11C,D). Although not significant, a small increase in IFN production was rather detected in pre- versus non-infected poly(I:C)-treated MEFs, reflecting most likely a contribution of the cytokine production triggered for 36 h by the parvovirus infection (see Figure 1B) to the one produced by poly(I:C) transfection (Figure 11D). Altogether, these data indicate that in contrast to the situation observed in transformed cells (see before), a rodent parvovirus infection of primary fibroblasts has no effect on the ability of classical inducers of antiviral mechanisms such as poly(I:C) to induce such a response.

**Figure 12:**
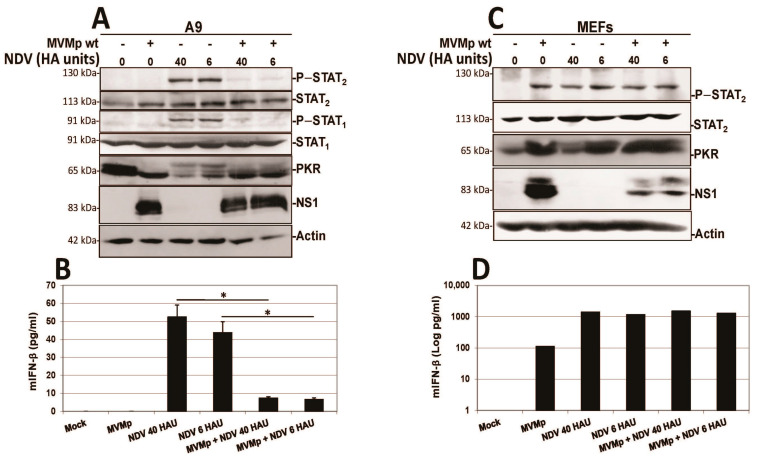
MVMp pre-infection of transformed (A9), but not of primary (MEF), mouse fibroblasts prevents further NDV-triggered IFN-β production. Cultures of transformed mouse A9 fibroblasts (**A**,**B**) or primary MEFs (**C**,**D**) were mock-treated or MVMp-infected (MOI of 15 pfu/cell) for 24 h. Cultures were then infected, or not, with NDV for 16 h before being processed for Western blotting (**A**,**C**). Cell-free culture media were harvested for IFN-β ELISA quantification (**B**,**D**). Results are presented as mean + SD of three (**B**) or two (**D**) independent experiments performed in duplicate. Statistical significance was calculated using a paired two-tailed *t* test: * *p* < 0.05.

Similar experiments were performed using as an antiviral response inducer the paramyxovirus NDV (Ulster strain 2C) instead of poly(I:C), a negative strand RNA virus triggering in both normal and transformed mammalian cells a potent IFN production [50,51,58]. As shown in Figure 12, here too we observed that the pre-infection of transformed A9 cultures by MVMp is able to significantly and almost fully prevent a further NDV-induced stimulation of an IFN-dependent response as well as IFN-β production (Figure 12A,B). As for the poly(I:C) treatment, NDV infection did not affect MVMp NS1 expression (Figure 12A) nor virus replication (Figure S11B), indicating that MVMp efficiently blocks the capacity of NDV to trigger an antiviral response in infected transformed cells. In MEF cultures, on the contrary, we observed something similar to the poly(I:C) transfection, that is, MVMp infections were unable to prevent the NDV-induced IFN-β signaling and production (Figure 12C,D).

Altogether, these experiments indicate that rodent parvovirus infections trigger a mechanism that blocks innate antiviral mechanisms more efficiently in transformed/tumor cells than in primary counterparts.

## 4. Discussion

Despite being questioned by some studies [59,60], we and others have previously shown that the infection of permissive and semi-permissive host cells with the autonomously replicating rodent protoparvoviruses MVMp and H-1PV triggers an antiviral innate immune response under both in vitro and in vivo conditions [40,41,54,61,62]. Interestingly, this response was observed in normal but not in transformed or malignant host cells. The anti-parvoviral cellular response involves the production of type-I IFNs and the induction of ISG expression through activation of the JAK–STAT pathway. In the present study, we provide new evidence indicating that while indeed failing to develop a full antiviral response upon PV infection, transformed cells nevertheless show activation of a PRR-dependent pathway. As in normal cells, both transcription factors IRF3 and NFκB become activated and translocate into the nucleus of transformed cells upon H1-PV or MVMp infection. Such events are hallmarks of the activation of an upstream PRR [7], indicating that although no IFN gene transcription and IFN production are detected in these cells, an innate antiviral pathway is nevertheless engaged. One could argue that such an observation simply confirms that transformed/neoplastic cells are intrinsically deficient in one or more factors belonging to, or modulating, IFN-β gene transcription, for instance, in the IFN-β enhanceosome activity [63]. This is, however, very unlikely since in most of the cell lines under investigation in our study, type-I IFN production and release can be induced by other PRR ligands such as poly(I:C) or by infection with the avirulent, lentogenic, NDV virus (Ulster 2C) that can partially evade RLR-dependent type-I IFN production only in avian cells but not in mammalian hosts [51]. Therefore, we speculate that as with many, if not all, natural viruses, the rodent parvoviruses MVMp and H-1PV have developed, along their co-evolution with their host, an innate immunity-evasion mechanism [24] that is triggered most efficiently in neoplastic cells. This strategy would allow the parvoviruses to block type-I IFN gene expression in transformed cells, thereby preventing development of an antiviral response and contributing to the known oncolytic activity of these agents. In agreement with this hypothesis, we provide data showing that pre-infection with MVMp or H-1PV of permissive transformed NB324K and HEK293T cells as well as malignant Hela cells, strongly prevents poly(I:C) transfection or NDV infection from triggering IFN-β production in these cells. This inhibition was not observed when normal cells (MEFs) were treated in the same way, confirming that parvoviral innate immune evasion does not function or is at least less efficient in non-transformed cells. Since this evasion mechanism is able to block both RLR-dependent (NDV infection or poly(I:C) transfection) and RLR-independent (parvovirus infection) IFN production, it appears to act at a step shared by several IFN-inducing pathways and one downstream of IRF3 and NFκB nuclear translocation. It is important to note here that feline panleukopenia virus (FPV), a feline protoparvovirus, was already reported to block IFN-β production in F81 feline cells [64]. However, in contrast to our observations, FPV was shown to act at a step prior to IRF3 nuclear translocation, namely, at its cytosolic phosphorylation/activation by TANK-binding kinase 1 (TBK1), an event common to all PRR-dependent pathways [65]. Such an effect was not observed in our study since IRF3 translocated into the nucleus in rodent PV-infected cells independently from their ability to produce IFN or not. The latter suggests that PVs may have evolved different and perhaps redundant strategies to block a step common to all innate immune pathways.

Nevertheless, since PVs replicate and are assembled in the nucleus, it may not be surprising that their immune evasion activities are associated with this organelle. The underlying molecular mechanisms as well as the parvoviral factors involved remain to be characterized. By analogy with the known properties of a number of virus-encoded ancillary/accessory proteins [66,67,68], it is tempting to speculate that the parvovirus ancillary NS2 proteins may be involved in the evasion process, as suggested by previous studies [64,69,70]. However, this hypothesis still needs to be experimentally validated. For example, while the NS2 protein of FPV was indeed identified as the parvoviral factor preventing Sendai virus-induced type-I IFN production [64], a recent study reported that the bovine parvovirus (BPV), genus *Bocavirus*, is able to block IFN production triggered by vesicular stomatitis virus (VSV) infections (i.e., activation of the RIG-I pathway), through its capsid VP1 polypeptides but not its NS2 proteins [71]. Notably, the amino acid sequence of the single FPV NS2 protein differs rather substantially (in particular, in its carboxy-terminal domain involved in TBK1 interaction [64]) from that of H-1PV and MVMp [72]. In addition, in contrast to FPV, both rodent PVs encode several isoforms of this non-structural polypeptide, suggesting that rodent and feline NS2 polypeptides may act at a different stage and in a different manner against IFN production pathways [73]. Altogether, our and other’s findings indicate that PV-induced evasion of innate immune responses explores diverse and complex mechanisms that may be triggered by different virus polypeptides depending on the particular PV type.

Another interesting finding from the present work is that the PRR triggering type-I IFN production upon PV infection of semi-permissive normal MEF cells is not TLR-9, in contrast to what was previously reported for abortively infected hPBMCs [41]. Indeed, TLR-9 knockout MEFs were as potent as their wild-type counterparts in IFN-β production upon MVMp infection. Furthermore, the fact that MAVS- and MyD88/TRIF-deficient primary MEFs produce similar cytokine amounts as wild-type cells upon rodent PV infection indicates that neither RLRs nor TLRs are involved in the activation of this antiviral response. The PRR(s) involved in rodent PV-induced type-I IFN production is (are) presently not known. It is noteworthy in this regard that in contrast to the situation encountered in hPBMCs, parvovirus replication is required for PRR activation in MEFs. A most likely alternative PRR candidate would thus consist in a DNA sensor linked to the adaptor protein STING, as already suggested by previous investigations using MVMp [62] or FPV [64], such as cGAS, IFI16, DDX41 or DNA-PK [19]. Since cGAS and IFI16 can both be present in the nucleus [18,74], these DNA sensors may indeed detect parvoviruses through their nuclear replication process and represent therefore the most likely candidates. The antiviral protein kinase R (PKR), which senses dsRNAs produced by many families of replicating viruses, was reported to efficiently restrict MVMp infection in permissive host cells [62,75,76]. This enzyme is, however, not involved in type-I IFN induction and can thus not be the PRR triggering the IFN pathway in PV-infected cells. The PKR gene belongs to the ISG family, whose transcription is upregulated upon the production and release of IFN by infected cells. Therefore, while not being involved in IFN production, PKR could still contribute to the anti-parvoviral response elicited by the cytokine in infected normal mouse fibroblasts. This possibility is supported by our observation that PKR gets activated through phosphorylation and is expressed at a higher level in MVMp-infected MEFs. PKR activation could thus constitute one arm of a global antiviral response triggered by parvovirus infection in normal cells upon upstream activation of a PRR-dependent type-I IFN production [75,76]. The PRR sensing H-1PV and MVMp infection remains, however, still to be identified.

Further findings of this study concern, on the one hand, the nuclear accumulation of dsRNA molecules in MVMp and H-1PV-infected permissive (transformed) as well as semi-permissive (normal) cells and, on the other hand, the observation, for the first time to our knowledge, of a correlation between the nuclear presence of these nucleic acids and the concomitant activation of transcription factors (i.e., IRF3 and NFκB nuclear translocation) belonging to the innate immune machinery. These features are thus not related to the host cell’s capacity for producing type-I IFN upon PV infection. Precedents can be found in reports of the ability of two members of the *Parvoviridae* family, human Bocavirus 1 (HBoV1) and MVMp, to trigger nuclear accumulation of dsRNA molecules in human (i.e., HEK293 and HEA) and mouse (i.e., A9) cells, respectively [56,77]. HBoV1-induced dsRNAs (called BocaSR) were found to be 140 nucleotide-long, to depend on RNA polymerase III for their transcription and to be required for parvoviral DNA replication. BocaSR shared sequence and structure homologies with adenovirus VAI RNAs, but in contrast to these RNAs, BocaSR did not act through PKR inhibition [78]. Interestingly, the infection of permissive cells with two negative-strand RNA viruses, Influenza A virus (IAV) and Nyamanini virus, also leads to the nuclear expression of dsRNAs [77] as expected from the mostly cytosolic location of the kinase [79]. Altogether, these data indicate that upon infection, several viruses, including at least some autonomously replicating parvoviruses, trigger the expression of potentially immunostimulatory molecules (i.e., dsRNA) in the nucleus of their host [80]. These observations raise the intriguing question of the role of these nuclear dsRNAs in the life cycle and more generally pathogenicity of these viruses. Given the strong homology observed between rodent parvovirus dsRNAs and BocaSR regarding (i) nucleotide sequence (over 55%), (ii) secondary structure, (iii) size (< 200 nts) and (iv) genome localization of the dsRNA-encoding region, it is tempting to speculate that MVMp/H-1PV dsRNAs also contribute, similar to BocaSR, to virus replication and multiplication. An argument in favor of this hypothesis resides in our observation of a nuclear colocalization between dsRNAs and NS1 in immunofluorescence experiments. The latter may thus suggest that interactions between NS1 and these dsRNA molecules may occur that could modulate, as with many other post-translational modifications, the multiple functions of this non-structural protein required for virus replication.

The origin of the dsRNA molecules accumulating in the nuclei of MVMp- and H-1PV-infected cells is presently a matter of speculation. In the case of HBoV1 infection, nuclear dsRNA (BocaSR) expression was assigned to a specific (terminal) non-coding region of the viral positive genome [56]. Our in silico analysis showed strong sequence and structure homology between BocaSR and a region located within the 5′ end of the MVMp and H-1PV negative genomes (SR region). It is therefore possible that as for HBoV1 infection, this segment of MVMp and H-1PV DNA is transcribed into the dsRNA molecules present in the nuclei of cells infected with these viruses. It should, however, be stated that while BocaSR expression proved to be driven by RNA polymerase III^47^, no obvious Pol III transcriptional hallmarks (promoter and termination sequences) were identified in the SR region of MVMp and H-1PV DNA. Whether the presence of NS1-binding and nicking sites in the latter region may contribute to its transcription into dsRNA remains to be investigated.

It is worth noting that BocaSR and rodent PV dsRNAs share structural features with endogenous Y RNAs [81], which were recently shown to act as PAMPS for RLRs (RIG-I/MDA-5) upon RNA virus infection [82]. This raises the question whether nuclear dsRNA molecules may play a role in the activation of the IFN pathway in MVMp- and H-1PV-infected cells. This possibility would be consistent with the hereby reported coincidence between nuclear dsRNA accumulation and nuclear translocation of the transcription factors NFkB and IRF3 in rodent PV-infected cells. The present study provided evidence that dsRNAs extracted from rodent PV-infected cells activate a type-I IFN production when introduced into the cytoplasm of naïve normal cells (MEFs) via transfection. This response depends on RLRs (RIG-I and/or MDA-5) and was not triggered in MAVs-deficient MEFs. It thus appears that MVMp- and H-1PV-induced dsRNAs have structural features allowing them to act as PAMPs when they are artificially transferred into the cytoplasm and brought into contact with cytosolic PRRs. It remains to be determined whether rodent PV-induced dsRNAs also fulfill a PAMP function when present in the nucleus, as detected in MVMp- and H-1PV-infected cells. A nuclear PRR able to sense dsRNA, called matrix protein SAFA and also known as HnRNPU, has recently been identified [83]. It is presently a matter of speculation whether rodent parvovirus PAMPs may consist of viral dsRNAs sensed by the latter type of receptor and/or viral DNA intermediates recognized by DNA sensors (e.g., cGAS, IFI16) which can also be present in the nucleus [18,74].

## 5. Conclusions

In conclusion, this study shows for the first time that the autonomously replicating rodent parvoviruses MVMp and H-1PV engage upon their replication, in both permissive (transformed/malignant) and semi-permissive (normal) cells, a PRR-dependent, type-I IFN-producing pathway. It further shows that both rodent PVs induce IFN production through IRF3 and NFκB activation and nuclear translocation, and that this induction is TLR- and RLR-independent. An alternative type-I IFN induction pathway may thus involve the PRR cGAS and its adaptor protein STING. This possibility is in keeping with recent reports indicating that STING contributes to MVMp attenuation in MEFs [62] and that the recruitment of TBK1 by STING is prevented by the NS2 protein of another protoparvovirus, FPV [64].

The PRR sensing H-1PV and MVMp infection remains, however, to be identified experimentally among the several upstream DNA sensors known to signal through STING. The present study raises, however, the intriguing possibility of additional PRR(s) playing a role in IFN induction by both rodent PVs.

This possibility is supported by two lines of evidence. First, we found that IRF3 and NFκB activation and nuclear transfer in H-1PV- and MVMp-infected cells correlates with the nuclear accumulation of viral dsRNAs endowed with PRR engagement capacity. Second, H-1PV- and MVMp-induced production of type-I IFN proved to be blocked in transformed cells at a late step of a PRR-dependent pathway engagement, following IRF3 and NFκB activation and nuclear migration. Therefore, at least some interaction(s) of H-1PV and MVMp factors with the IFN production machinery appear(s) to take place in the nucleus of infected cells. These observations lead us to speculate that some of the receptors recognizing parvoviral PAMPs may be located in the nucleus. Further work is now required to confirm the identity and role of these putative nuclear PRR(s).

Moreover, our study also reveals that in PV-permissive cells, the antiviral response is very efficiently aborted prior to IFN production through an innate immune evasion mechanism allowing rodent PVs to escape the antiviral effects of interferons. Further investigations are now needed to characterize the molecular mechanisms underlying the intracellular sensing of rodent PVs and the capacity of these viruses for modulating innate immune responses in both positive and negative ways. These investigations are relevant not only from the academic but also from the applied point of view. Indeed, rodent PVs are presently developed in the framework of cancer oncolytic virotherapy [84], with H-1PV being in particular the subject of two recent clinical studies in cancer patients [37,38]. Besides direct viral oncolysis, the mobilization of the immune system against tumors represents an essential component of the viral therapeutic strategy [85]. A better understanding of the up- and down-modulating effects of rodent PVs on immune responses is hence important to optimize the efficiency of cancer treatments based on these agents. This can be exemplified by the case of type-I IFNs, which are endowed with an immunostimulating capacity and whose production in the TME indicates a good prognosis after various cancer treatments [32,86,87,88,89]. Given the reported lack of sensitivity of rodent PVs to the antiviral effects of type-I IFNs [60], virus modifications impairing the abovementioned innate immune evasion process may confer enhanced oncosuppressive properties as a result of increased type-I IFN production by infected tumor cells and ensuing stimulation of anticancer immune reactions. This effect may be exacerbated by the release of dsRNA molecules from dying PV-infected tumor cells and the activation of the neighboring immune cells, which would have taken up these PAMPs.

## Data Availability

Data are available on request from the corresponding author.

## Supplementary Materials

The following supporting information can be downloaded at https://www.mdpi.com/article/10.3390/pathogens12040607/s1, Figure S1: Mouse transformed fibroblasts are endowed with functional PRR-dependent type-I IFN production pathways, Figure S2: MVMp infection of mouse melanoma cells induces NFĸB and IRF3 nuclear translocation and activation, Figure S3: Parvovirus infection of human NB324K and cervical cancer cells induces nuclear translocation and activation of NFĸB and IRF3, Figure S4: UV-inactivated MVMp particles are as infectious as their non-irradiated wild type counterparts, Figure S5: A proportion of parvovirus-infected cells displays nuclear expression of both dsRNA and NS1, Figure S6: Nuclear dsRNA production is detected in both MVMp- and H-1PV-infected transformed human cells, Figure S7: Cytoplasmic expression of dsRNA and NS1 is detected in a small proportion of parvovirus-infected cells, Figure S8: Methods of RNA isolation display variable abilities to isolate PV-produced dsRNA, Figure S9: UNAFold—based secondary RNA structure predictions for BocaSR, Figure S10: The 3’ extremity of the H-1PV genome contains a region that shares high sequence homology with the putative MVMpSR, Figure S11: (A) Parvovirus pre-infection of human transformed cell cultures prevents further poly(I:C)-triggered IFN-β production and (B) Parvovirus MVMp replication is not inhibited by NDV infection.
