# Supplementary material for: Oncolytic Rodent Protoparvoviruses Evade a TLR- and RLR-Independent Antiviral Response in Transformed Cells
Source: Pathogens. 2023 Apr 17;12(4):607. doi: 10.3390/pathogens12040607 (PMC10144674; doi:10.3390/pathogens12040607)
Supplement: Supplementary file 1 [file pathogens-12-00607-s001.zip › pathogens-2279962-Legends to Supplementary figures.pdf]

Supplementary Materials

Figure S1: Mouse transformed fibroblasts are endowed with functional PRR-dependent type-I IFN production pathways. Primary (MEFs) and transformed (A9) mouse fibroblasts were mock-treated or infected with NDV (40 HAU/10^6 cells). Mouse IFN-β was measured in culture supernatants 16h after the infection. Data are presented as mean + SD of three independent experiments. nd: not detected.

Figure S2: MVMp infection of mouse melanoma cells induces NFκB and IRF3 nuclear translocation and activation. B78/H-1 cells were mock-treated or infected with MVMp (MOI 5 pfu/cell) for 48h.NS1 (red) and NFκB or IRF3 (green) were visualized using indirect immunofluorescence. Nuclei were counterstained using Hoechst staining. Scale bar: 20 µm.

Figure S3: Parvovirus infection of human NB324K and cervical cancer cells induces nuclear translocation and activation of NFκB and IRF3. (A) NB324K cells were either mock-treated or infected with MVMp (MOI 20 pfu/cell) or H-1PV (20 pfu/cell) for 30h. NS1 (red), NFκB (upper panel, green) and IRF3 (lower panel, green) were visualized using indirect immunofluorescence. (B) HeLa cells were either mock-treated or MVMp-infected (MOI 10 pfu/cell) NS1 (red), NFκB (upper panel, green) and IRF3 (lower panel, green) were analysed using indirect immunofluorescence.

Figure S4: UV-inactivated MVMp particles are as infectious as their non-irradiated wild type counterparts. Transformed mouse A9 fibroblasts were either mock-treated or infected with equal doses of wild type versus UV-inactivated MVMp particles corresponding for the former virus type to an Moi of 30 pfu/cell.Cells were stained against (A) NS1 (red) [43] or (B) intact MVMp capsids (green) and lamin B (red). Scale bar: 20 µm.

Figure S5: A proportion of parvovirus-infected cells displays nuclear expression of both dsRNA and NS1. (A) Primary MEFs and transformed mouse A9 fibroblasts were infected for 24h with MVMp at 10 pfu/cell. and analysed by indirect immunofluorescence using dsRNA (green)- and NS1 (red)-specific antibodies. (B) MVMp-infected primary MEFs and transformed mouse A9 fibroblastswere infected with MVMp at 10 pfu/cell for 30h before being analysed by indirect immunofluorescence using dsRNA (green)- and lamin B (red)-specific antibodies. (C) Primary MEFs and transformed mouse A9 fibroblasts were transfected with poly(I:C). Expression of dsRNA (green) and lamin B (red) was analysed by indirect immunofluorescence. (D) dsRNA (green) and NS1 (red) expression in MVMp-infected B78 mouse melanoma cells infected for 48h with MVMp at 10 pfu/cell was analysed by indirect immunofluorescence.

Figure S6: Nuclear dsRNA production is detected in both MVMp- and H-1PV-infected transformed human cells. Transformed human newborn kidney NB324K (A, B) and human embryonic kidney HEK293 (C) cells were either mock-treated or infected with MVMp (5 and 10 pfu/cell, respectively) or H-1PV. (2 and 10 pfu/cell, respectively) for 36h.Colocalization of (A, C) dsRNA (green) and NS1 (red), or (B) dsRNA (green) and lamin B (red) expression was analysed using indirect immunofluorescence.

Figure S7: Cytoplasmic expression of dsRNA and NS1 is detected in a small proportion of parvovirus-infected cells. (A, B) Transformed mouse A9 fibroblasts were infected for 24h with MVMp (10 pfu/cell) or with NDV (40 HAU/10^6 cells) for 16h Samples were analysed by indirect immunofluorescence using (A) dsRNA- and (B) both dsRNA- and NS1-specific antibodies. (C) Primary MEFs were infected with MVMp and stained against dsRNA and NS1. Scale bar: 20 µm.

Figure S8: Methods of RNA isolation display variable abilities to isolate PV-produced dsRNA. Primary wild type MEFs were transfected with 1 µg/ml total RNA extracted 48h post mock-treatment or parvovirus infections (MOI 1 pfu/cell) of transformed mouse A9 fibroblasts. Total RNA was isolated either by Trizol extraction or according to the Qiagen RNeasy kit protocol from the same infected culture. IFN-β release in the cell-free medium was quantified by ELISA. Data are presented as mean + SD of three independent experiments performed in duplicate.

Figure S9: UNAFold—based secondary RNA structure predictions for BocaSR. Predicted secondary structures of BocaSR based on the sequence previously published[54] using the UNAFold algorithm[85]. For each structure, a thermodynamic value reflecting its potential stability is indicated.

Figure S10: The 3′ extremity of the H-1PV genome contains a region that shares high sequence homology with the putative MVMpSR. (A) SnapGene 6.0.5-based alignment between the sequence of MVMpSR and the full H-1PV (NCBI accession # X01457.1). Numbers indicate the position of nucleotides of each sequence that were aligning. Black dots refer to nucleotides that differ between each DNA sequence. Blue lanes correspond to NS1 binding domains (ACCA/TGGT motif) within the MVMp and H-1PV sequences while the purple lanes correspond to NS1 nick-site. (B) Predicted secondary structures of MVMpSR using the UNAFold algorithm [85].

Figure S11: (A) Parvovirus pre-infection of human transformed cell cultures prevents further poly(I:C)-triggered IFN-β production. Human transformed NB324K, HeLa, HEK293 and HEK293T cells were mock-treated or infected with either MVMp (MOI of 2, 10, 10 and 5 pfu/cell, respectively) or H-1PV (MOI of 2, 10, 5 and 5 pfu/cell, respectively) for 24h. Cultures were then transfected or not with the synthetic dsRNA poly(I:C). Cell-free culture medium was harvested and human IFN-β was quantified by ELISA. Data are presented as means of two independent experiments. (**B) Parvovirus MVMp replication is not inhibited by NDV infection.** Transformed mouse A9 fibroblasts were mock-treated or MVMp-infected (MOI of 10 pfu/cell) for 24h. Cultures were then super-infected or not with NDV (6 or 40 HAU/10^6 cells) for 16h.Total DNA was extracted and analysed by Southern blot experiments using a ^{32}P-labeled DNA probe corresponding to nucleotides 385–1885 of the NS1-encoding region of MVMp DNA. dRF, dimeric replicative form; mRF, monomeric replicative form; ssDNA, single-stranded DNA genome. Data are representative of three independent experiments showing similar results.
