# Supplementary material for: Oncolytic Rodent Protoparvoviruses Evade a TLR- and RLR-Independent Antiviral Response in Transformed Cells
Source: Pathogens. 2023 Apr 17;12(4):607. doi: 10.3390/pathogens12040607 (PMC10144674; doi:10.3390/pathogens12040607)
Supplement: Supplementary file 1 [file pathogens-12-00607-s001.zip › Revised supplementary Figures.pdf]

Figure S1

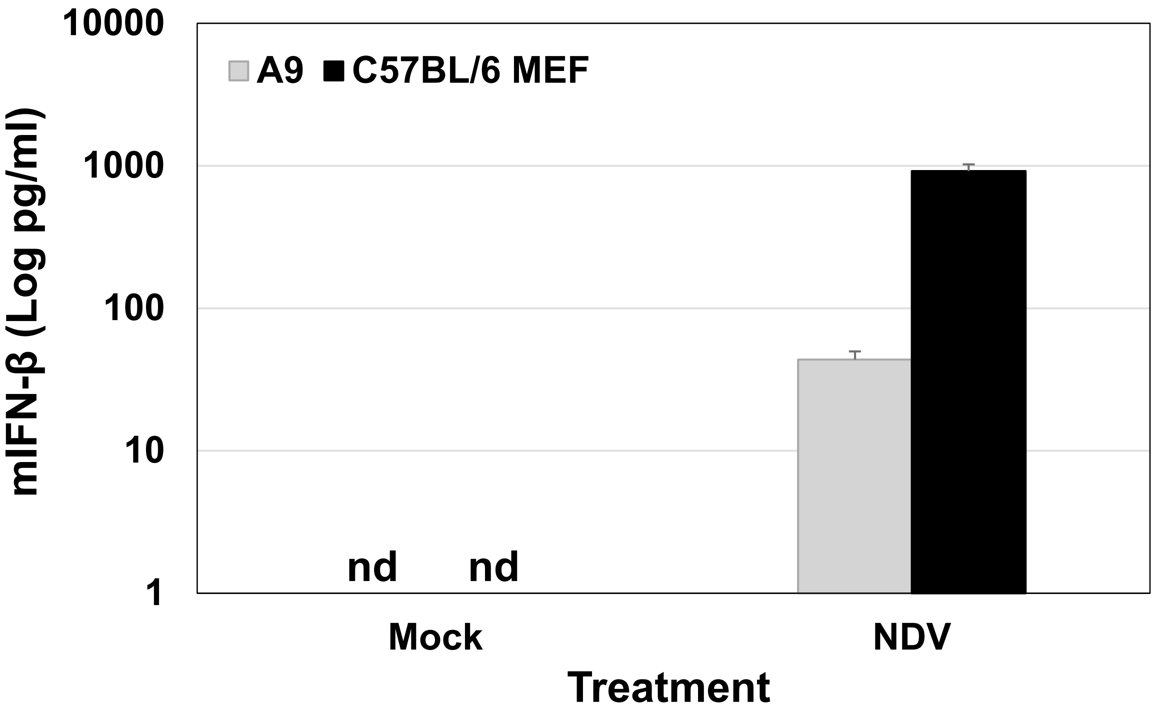

A9
C57BL/6 MEF
mIFN-β (Log pg/ml)
10000
1000
100
10
1
nd
nd
Mock
NDV
Treatment

Figure S2

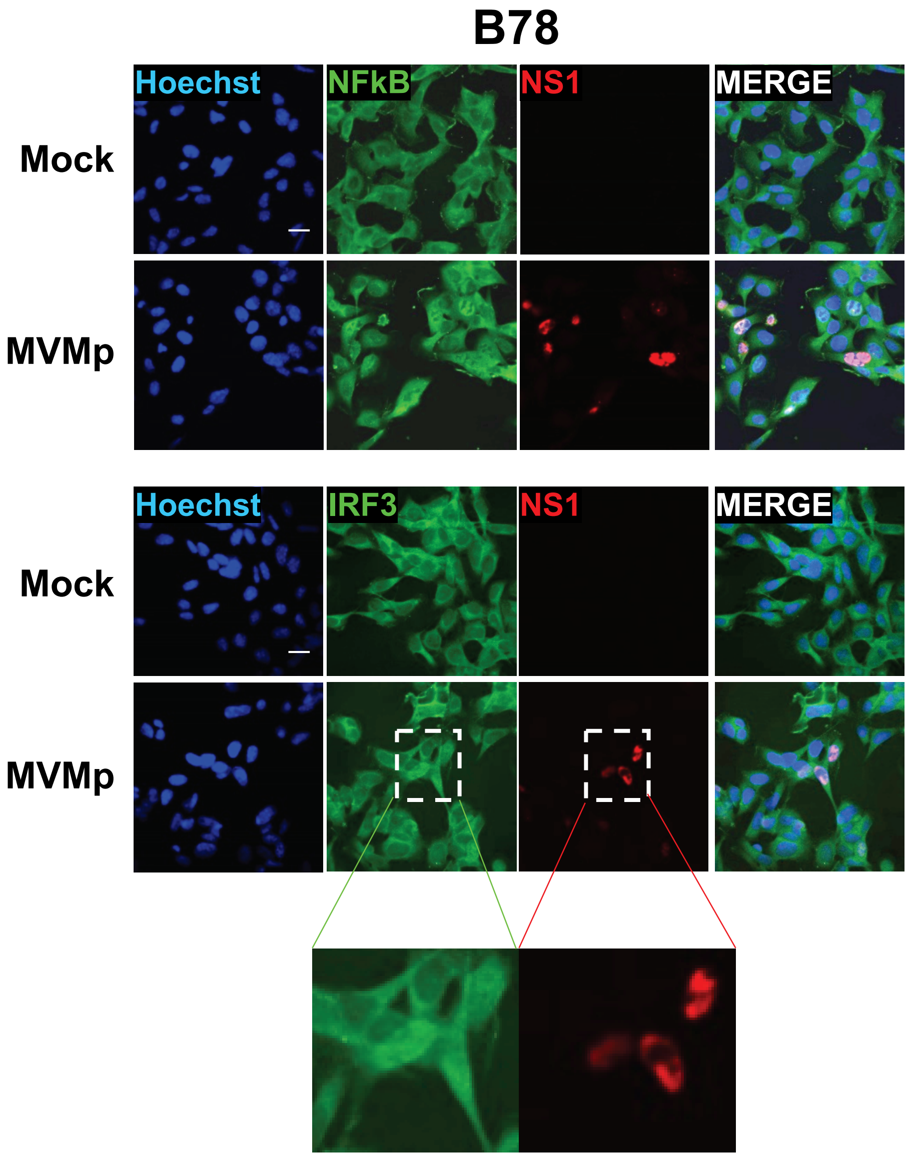
B78
Hoechst
NFkB
NS1
MERGE
Mock
MVMp
Hoechst
IRF3
NS1
MERGE
Mock
MVMp

Figure S3

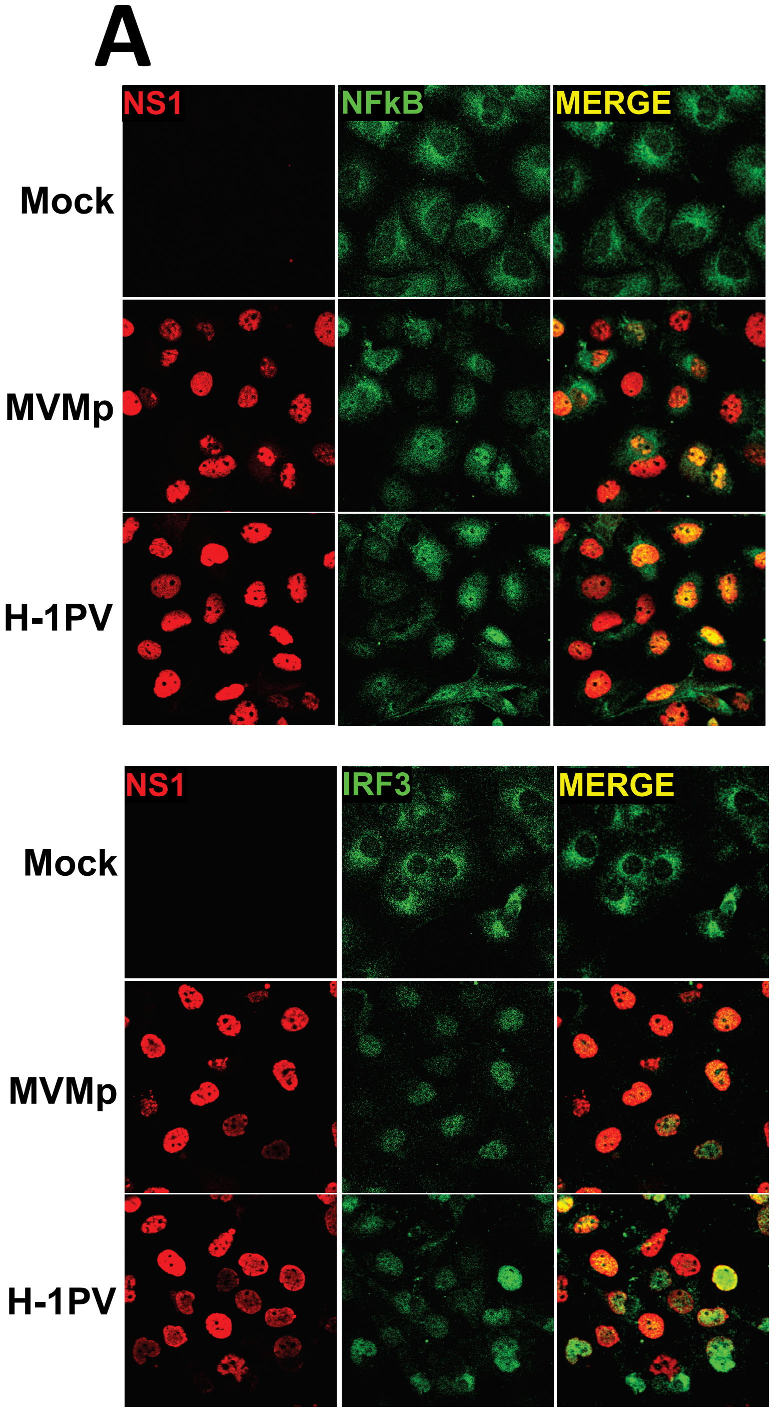
A
NS1
NFkB
MERGE
Mock
MVMp
H-1PV
NS1
IRF3
MERGE
Mock
MVMp
H-1PV

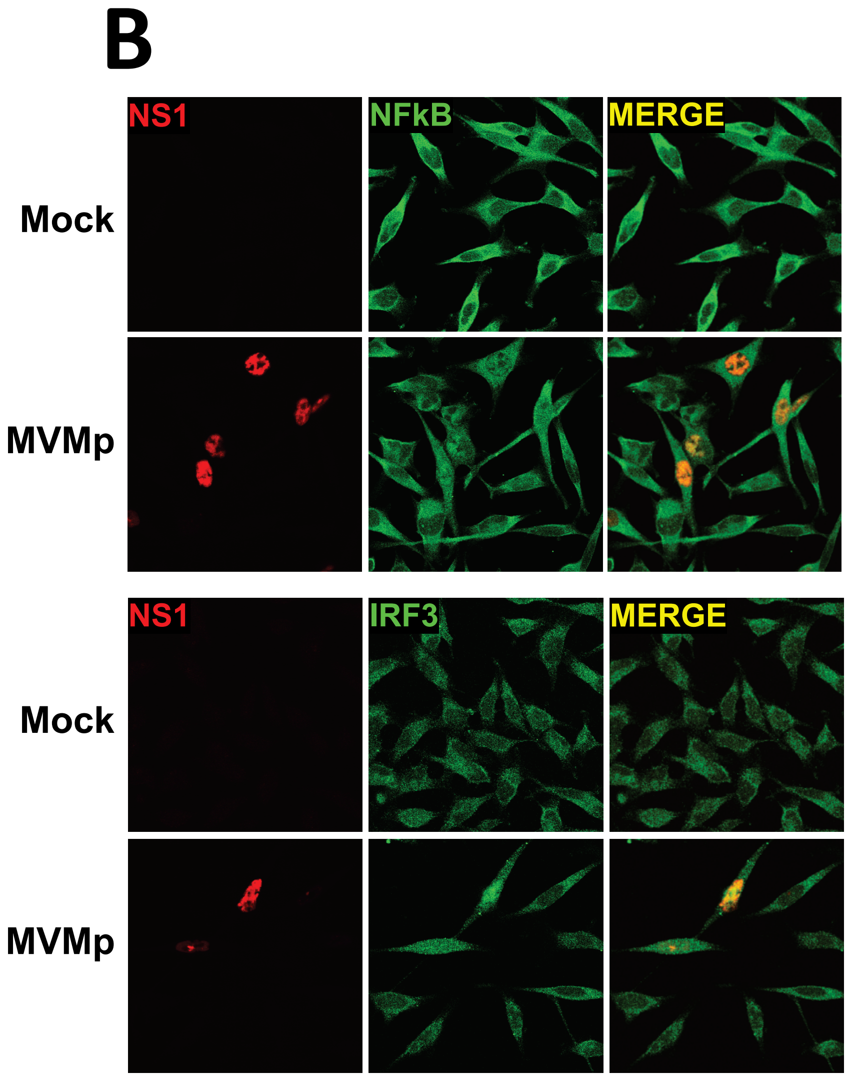
B
NS1
NFkB
MERGE
Mock
MVMp
NS1
IRF3
MERGE
Mock
MVMp

Figure S4

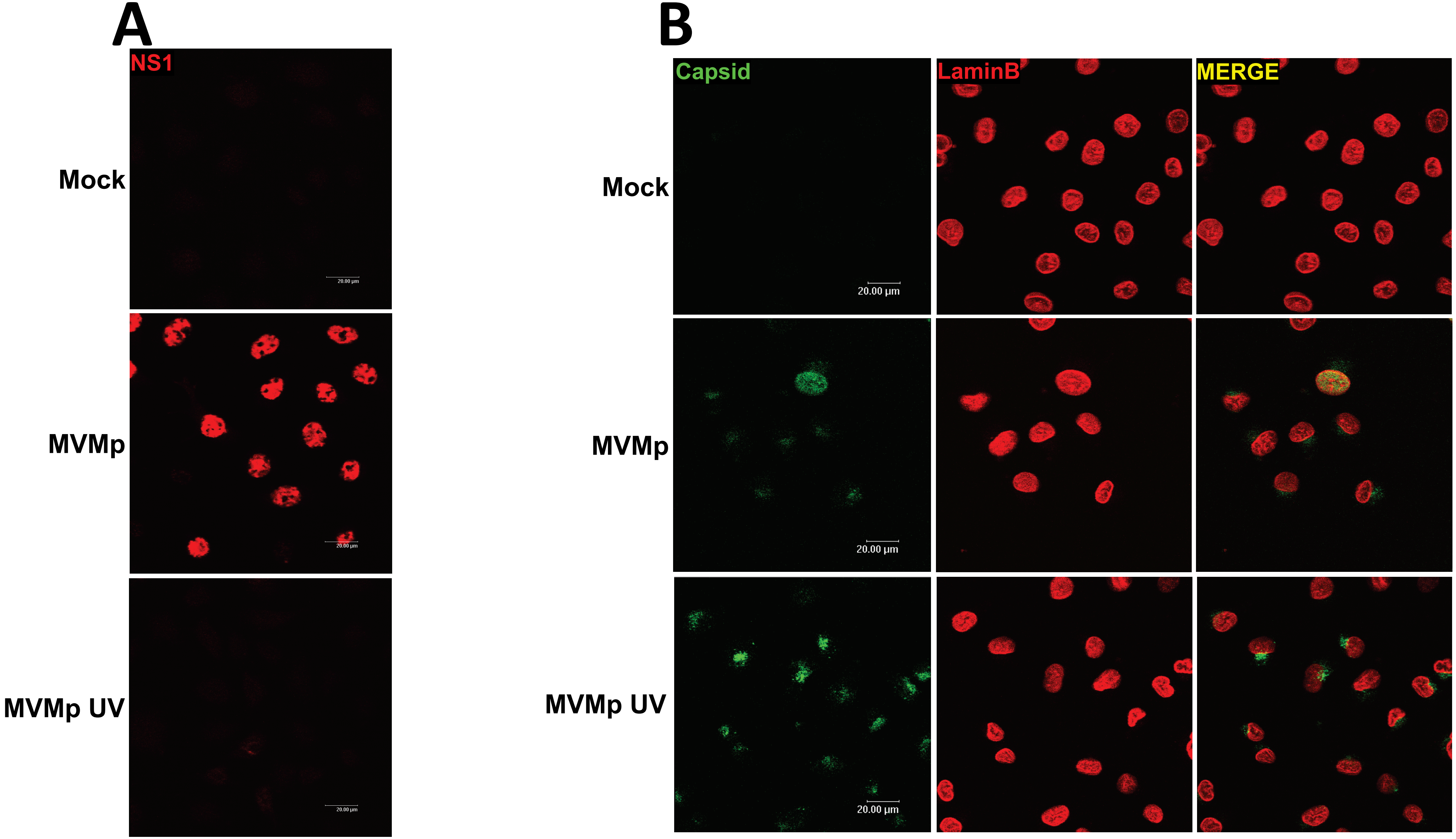

A
NS1
Mock
MVMp
MVMp UV
B
Capsid
LaminB
MERGE
Mock
MVMp
MVMp UV
20.00 µm
20.00 µm
20.00 µm

Figure S5

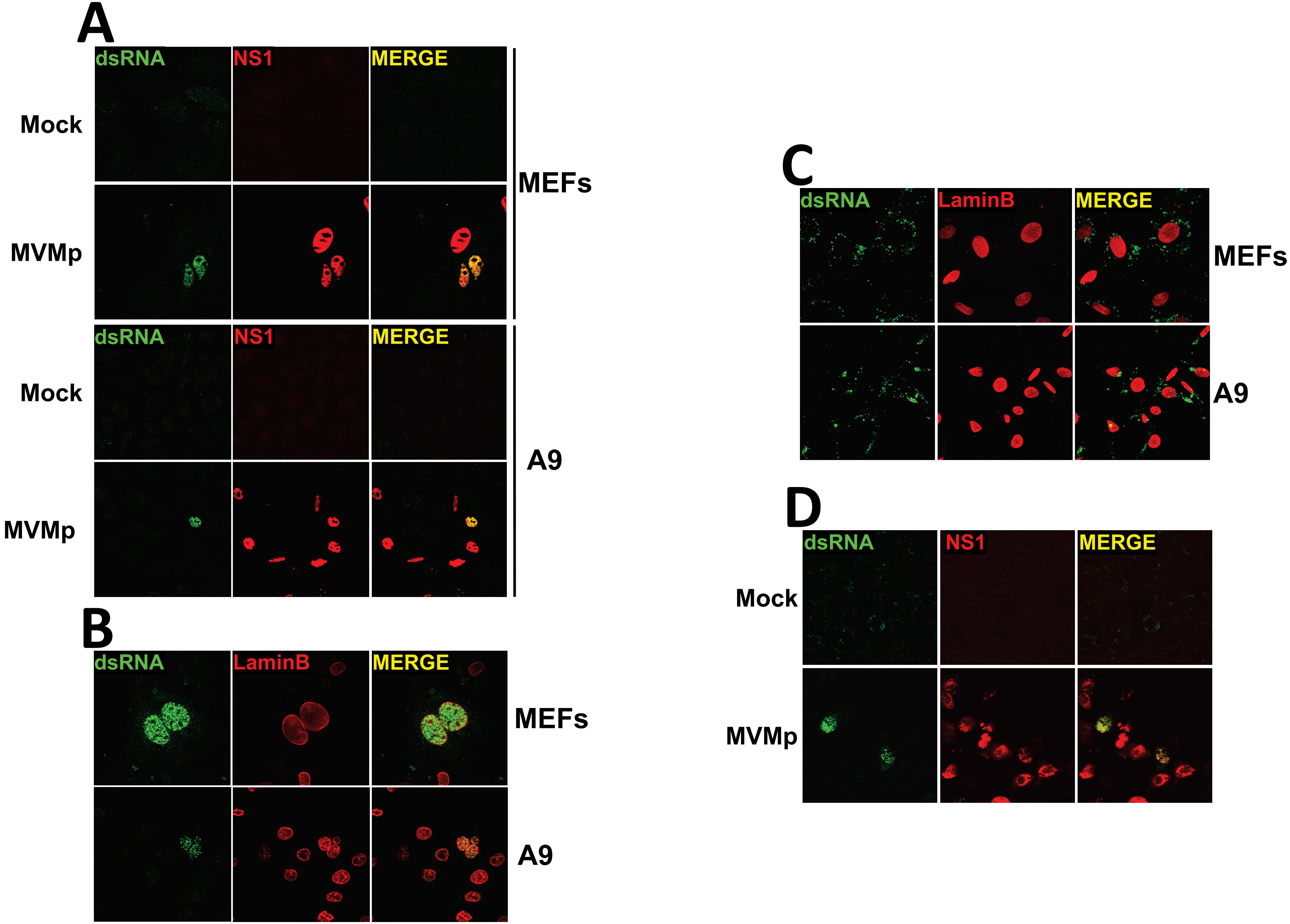
A
dsRNA
NS1
MERGE
Mock
MVMp
MEFs
dsRNA
NS1
MERGE
Mock
MVMp
A9
B
dsRNA
LaminB
MERGE
MEFs
A9
C
dsRNA
LaminB
MERGE
MEFs
A9
D
dsRNA
NS1
MERGE
Mock
MVMp

Figure S6

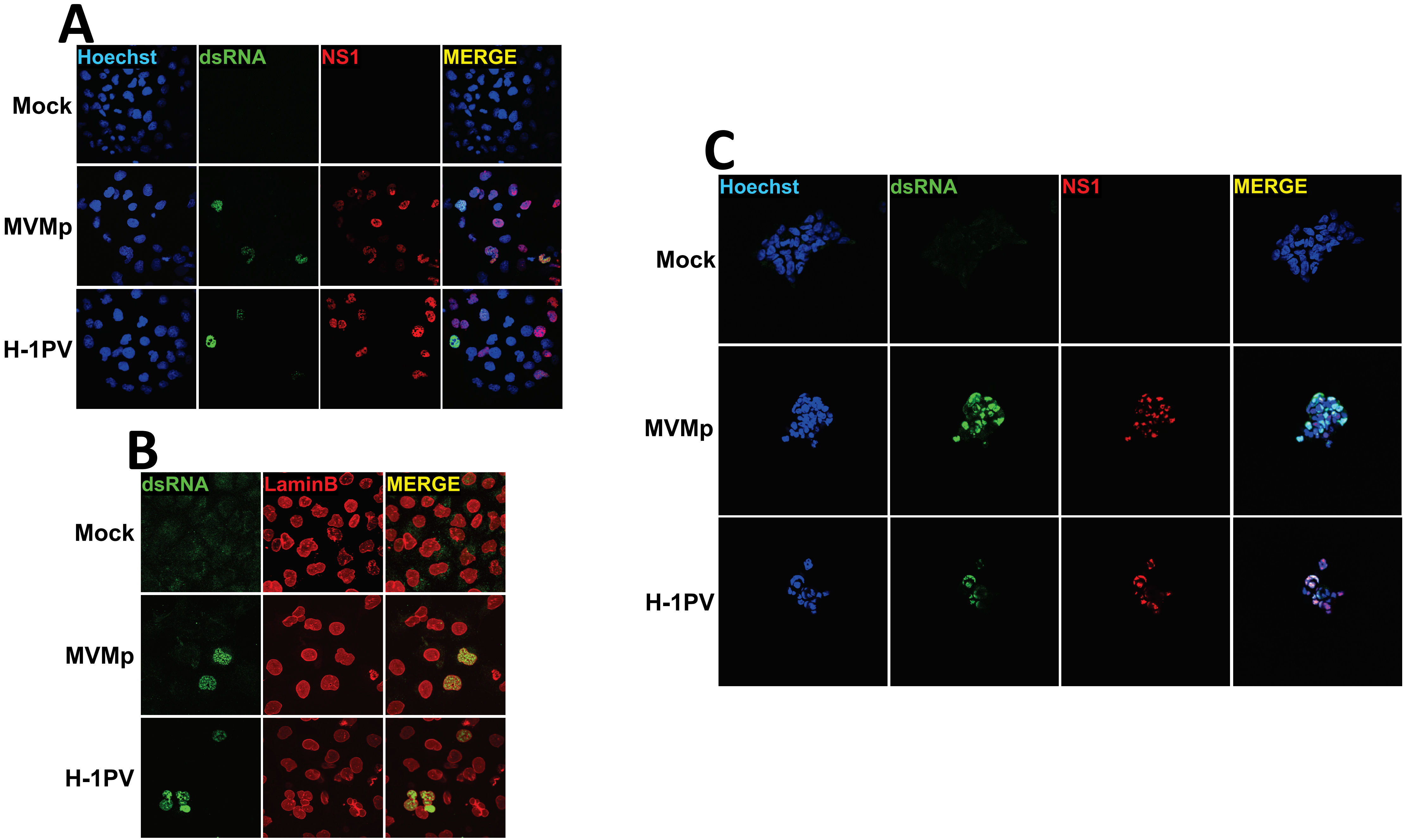
A
Hoechst
dsRNA
NS1
MERGE
Mock
MVMp
H-1PV
B
dsRNA
LaminB
MERGE
Mock
MVMp
H-1PV
C
Hoechst
dsRNA
NS1
MERGE
Mock
MVMp
H-1PV

Figure S7

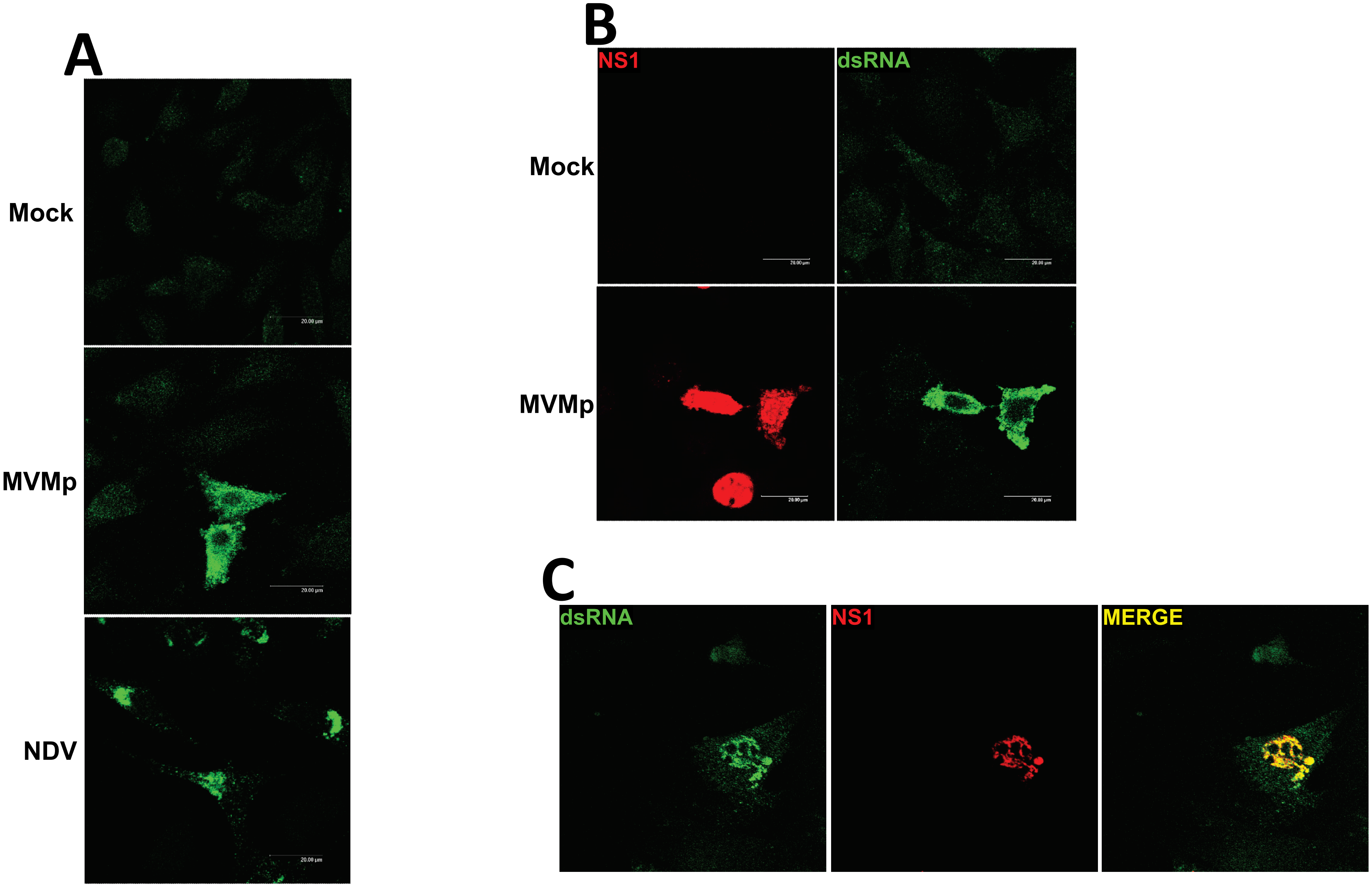

Figure S8

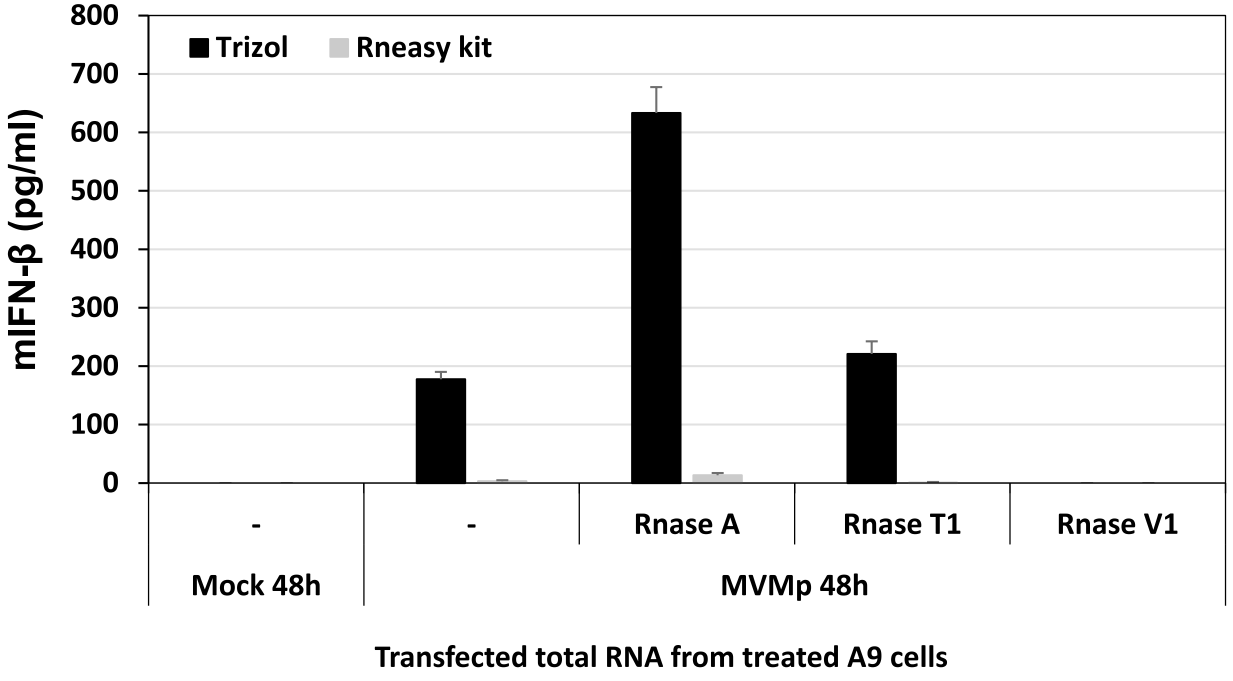

800
700
600
500
400
300
200
100
0
Trizol
Rneasy kit
mIFN-β (pg/ml)
-
-
Rnase A
Rnase T1
Rnase V1
Mock 48h
MVMp 48h
Transfected total RNA from treated A9 cells

Figure S9

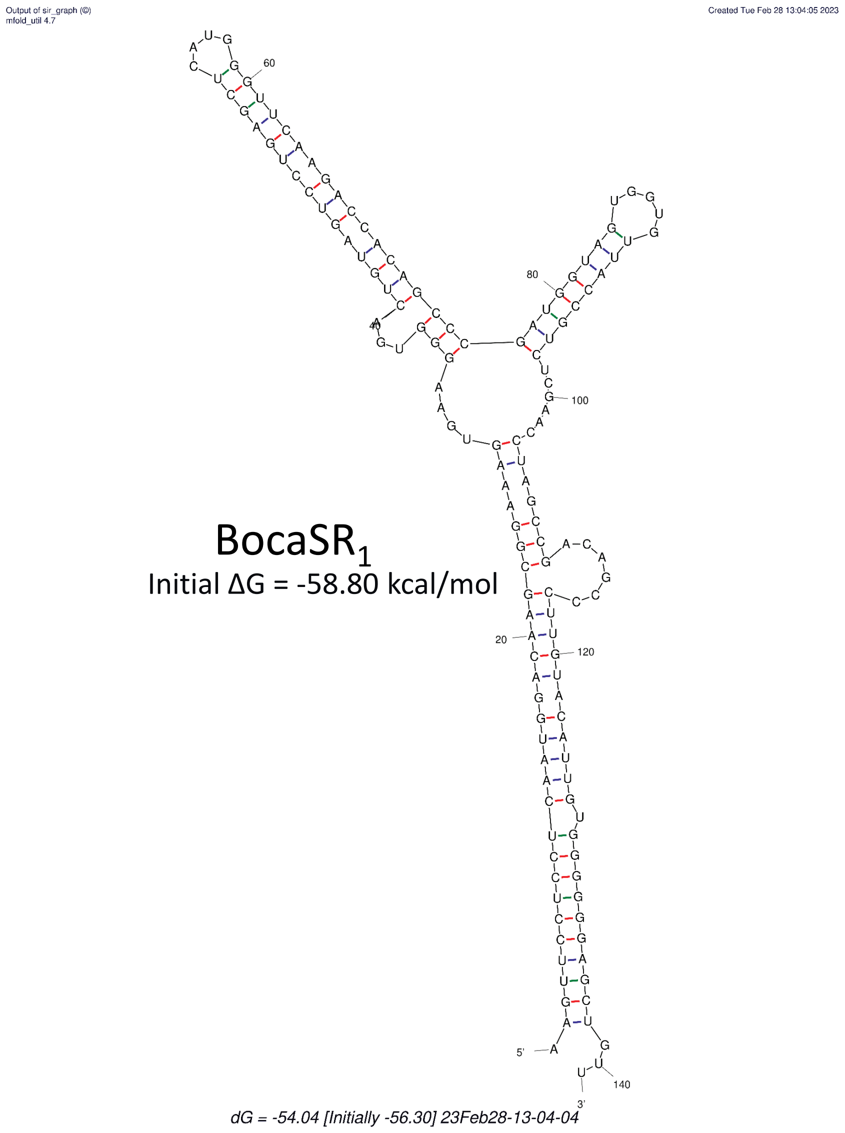

BocaSR$_1$
Initial ΔG = -58.80 kcal/mol

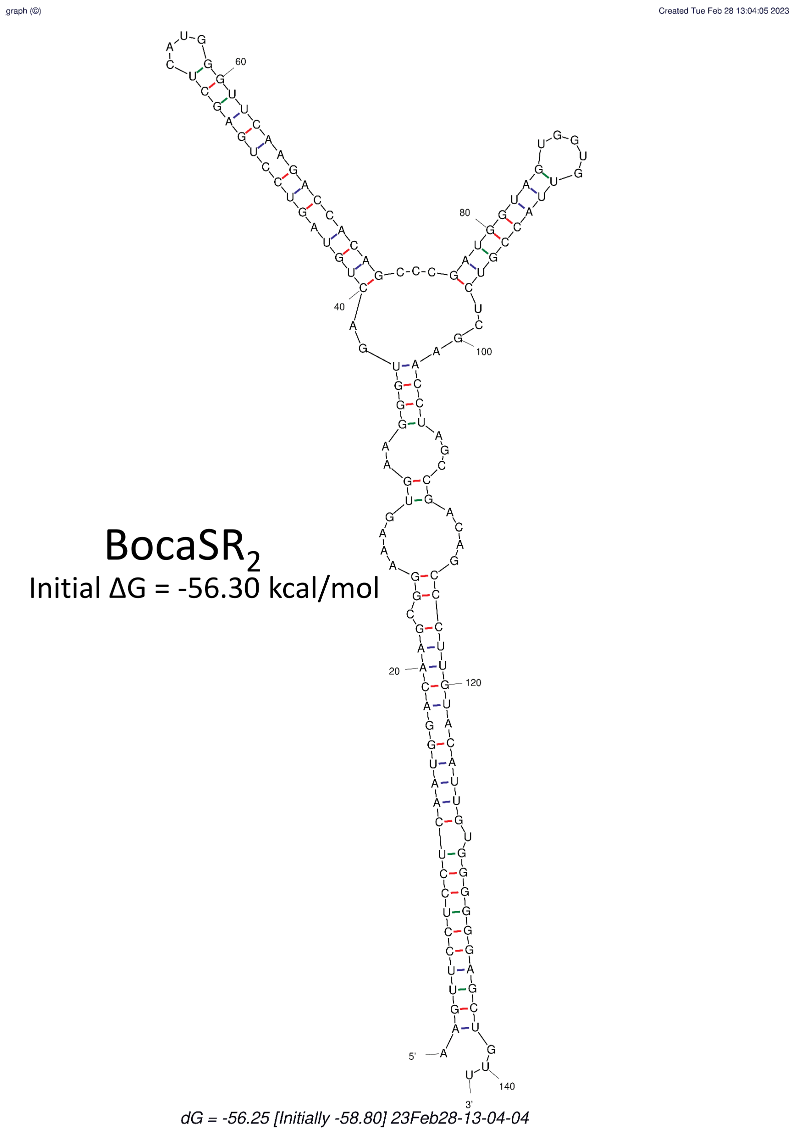

BocaSR$_2$
Initial ΔG = -56.30 kcal/mol

Figure S10

A

```
           1                                                                                                                                                    149
MVMpSR     CTATTCAGTGAACCAACTGAACCATTAGTATTACTATGTTTTTAGGGTGGGAGGGTGGGAGATACATGTGTTCGCTATGAGCGAACTGGTACTGGTTGGTTGCTCTGCTCAACCAACCAGACCGGCAAAGCCGGTCTGGTTGGTTGAGC
H-1PV      CTATTCAGTTGACCAACTGAACCTATAGTATCACTATGTTTTTAGGGTGGGGGGGTGGGAGATACATACGTTCGCTATGGACCAAGTGGTACCGGTTGGTTGCTAAGCT---CGAACAAGA-CGGCTAAGCCGGTCCGGTTGGTTGAGC
           4932                                                                                                                                                 5076
```

B

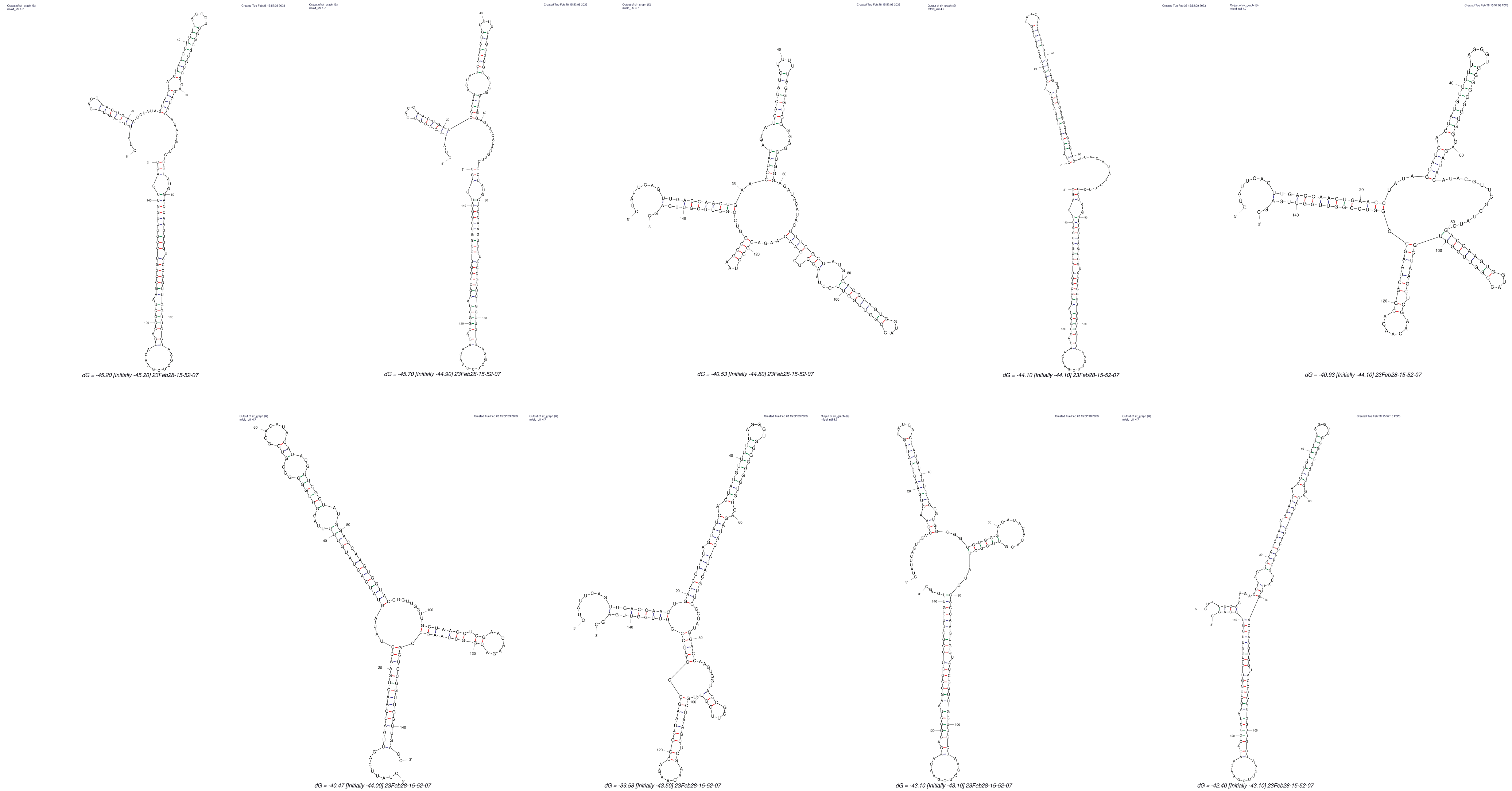

Figure S11

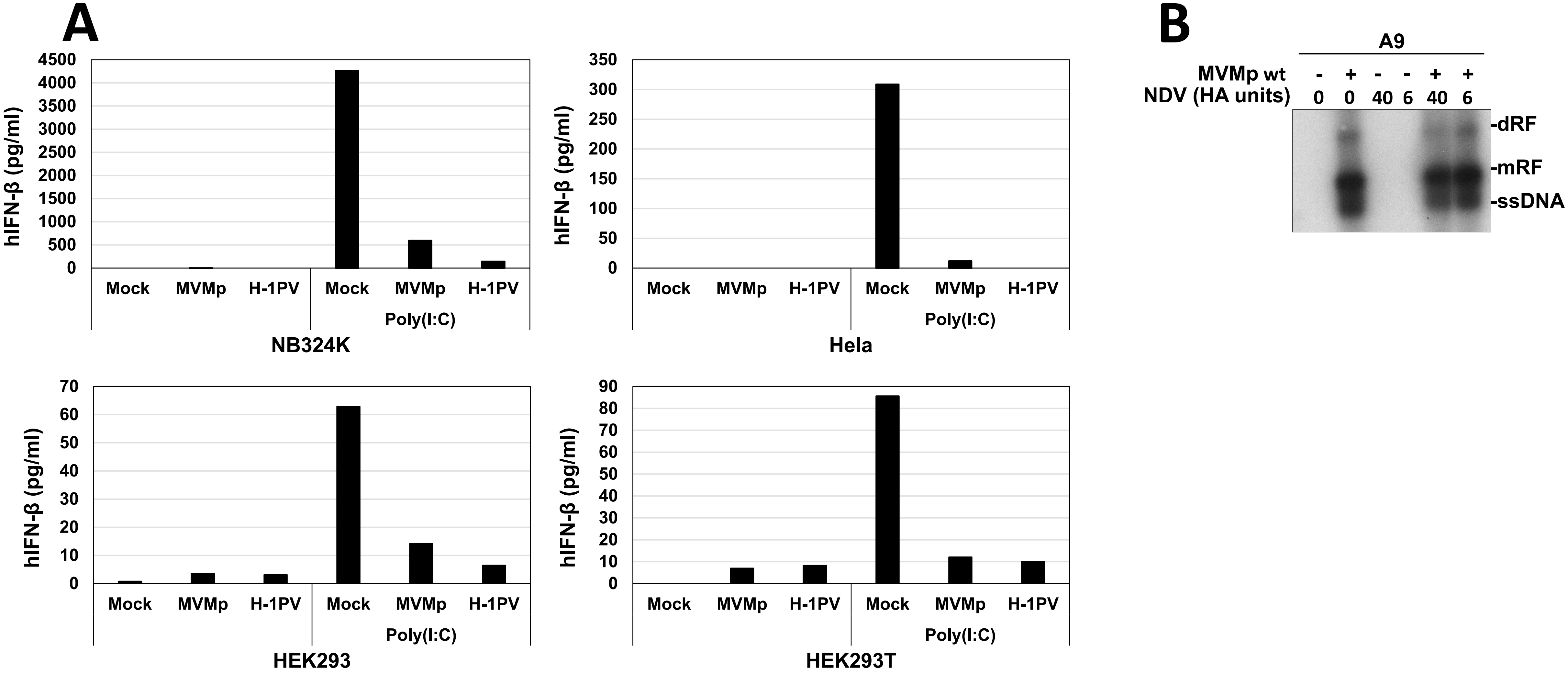
A
hIFN-β (pg/ml)
4500
4000
3500
3000
2500
2000
1500
1000
500
0
Mock
MVMp
H-1PV
Mock
MVMp
H-1PV
Poly(I:C)
NB324K
350
300
250
200
150
100
50
0
Hela
70
60
50
40
30
20
10
HEK293
90
80
HEK293T
B
A9
MVMp wt
- + - - + +
NDV (HA units)
0 0 40 6 40 6
dRF
mRF
ssDNA
